# Molecular basis of dual anti-CRISPR and auto-regulatory functions of AcrIF24

**DOI:** 10.1093/nar/gkac880

**Published:** 2022-10-16

**Authors:** Gi Eob Kim, So Yeon Lee, Nils Birkholz, Kotaro Kamata, Jae-Hee Jeong, Yeon-Gil Kim, Peter C Fineran, Hyun Ho Park

**Affiliations:** College of Pharmacy, Chung-Ang University, Seoul 06974, Republic of Korea; Department of Global Innovative Drugs, Graduate School of Chung-Ang University, Seoul 06974, Republic of Korea; College of Pharmacy, Chung-Ang University, Seoul 06974, Republic of Korea; Department of Global Innovative Drugs, Graduate School of Chung-Ang University, Seoul 06974, Republic of Korea; Department of Microbiology and Immunology, University of Otago, PO Box 56, Dunedin 9054, New Zealand; Bioprotection Aotearoa, University of Otago, PO Box 56, Dunedin 9054, New Zealand; Department of Microbiology and Immunology, University of Otago, PO Box 56, Dunedin 9054, New Zealand; Bioprotection Aotearoa, University of Otago, PO Box 56, Dunedin 9054, New Zealand; Pohang Accelerator Laboratory, Pohang University of Science and Technology, Pohang 37673, Republic of Korea; Pohang Accelerator Laboratory, Pohang University of Science and Technology, Pohang 37673, Republic of Korea; Department of Microbiology and Immunology, University of Otago, PO Box 56, Dunedin 9054, New Zealand; Bioprotection Aotearoa, University of Otago, PO Box 56, Dunedin 9054, New Zealand; College of Pharmacy, Chung-Ang University, Seoul 06974, Republic of Korea; Department of Global Innovative Drugs, Graduate School of Chung-Ang University, Seoul 06974, Republic of Korea

## Abstract

CRISPR-Cas systems are adaptive immune systems in bacteria and archaea that provide resistance against phages and other mobile genetic elements. To fight against CRISPR-Cas systems, phages and archaeal viruses encode anti-CRISPR (Acr) proteins that inhibit CRISPR-Cas systems. The expression of *acr* genes is controlled by anti-CRISPR-associated (Aca) proteins encoded within *acr-aca* operons. AcrIF24 is a recently identified Acr that inhibits the type I-F CRISPR-Cas system. Interestingly, AcrIF24 was predicted to be a dual-function Acr and Aca. Here, we elucidated the crystal structure of AcrIF24 from *Pseudomonas aeruginosa* and identified its operator sequence within the regulated *acr-aca* operon promoter. The structure of AcrIF24 has a novel domain composition, with wing, head and body domains. The body domain is responsible for recognition of promoter DNA for Aca regulatory activity. We also revealed that AcrIF24 directly bound to type I-F Cascade, specifically to Cas7 via its head domain as part of its Acr mechanism. Our results provide new molecular insights into the mechanism of a dual functional Acr-Aca protein.

## INTRODUCTION

As a result of the battle between bacteria and their invaders, including bacteriophages (phages) and other mobile genetic elements (MGEs), bacteria have evolved diverse defense mechanisms (1). In response, phages have developed anti-defense systems that work to suppress bacterial defense (2). Clustered Regularly Interspaced Short Palindromic Repeats (CRISPRs) and CRISPR-associated proteins (Cas) form CRISPR-Cas systems that provide adaptive immunity against invading genetic material (3–6). CRISPR-Cas systems are adaptive since they ‘record’ memories of past infections within their CRISPR arrays to elicit a rapid immune response to subsequent infections (7). CRISPR-Cas systems function via three distinct stages: adaptation, expression and interference (8,9). In the adaptation stage, invading DNAs are processed and integrated as spacers into a CRISPR array within the bacterial genome. Next, during the expression stage, the host CRISPR array is transcribed and processed into small CRISPR RNAs (crRNAs). Finally, in the interference stage, crRNA-guided complexes recognize crRNA-complementary sequences in invaders and either act alone or recruit additional proteins to cleave DNA or RNA of the invader (9–11). Due to the ability of specific DNA cleavage by CRISPR-Cas systems, they have been used for gene editing and their application on disease treatments is being tested (12–14).

The long evolutionary interaction between bacteria and phages has led to the diversification of CRISPR-Cas systems, which are currently grouped into two broad classes (class 1 and class 2) encompassing six types (type I to type VI) based on the CRISPR locus organization, the *cas* gene composition, and their mechanisms (15). The class 1 systems, including types I, III, and IV, employ multi-subunit Cas proteins for performing multiple functions, whereas class 2 systems, including types II, V, and VI, utilize a single multi-domain Cas protein containing all necessary activities (15).

The type I CRISPR-Cas systems are the most abundant and widely distributed, and are classified into seven subtypes, I-A through I-G, according to their signature *cas* genes and composition of Cas components (15). Multiple Cas proteins in type I systems associate with crRNA to form a CRISPR-associated complex for antiviral defense (Cascade) that recognizes invader DNA and recruits Cas3, the nuclease responsible for destroying target DNA (11).

To counteract these anti-phage immune systems, phages have evolved systems to aid in their evasion of these immune systems (2). One of the most well-characterized evasion strategies in phages is to encode anti-CRISPR proteins (Acrs) that can neutralize the host CRISPR-Cas system (16). Since the first Acrs were discovered in phages capable of blocking the type I-F CRISPR-Cas system of *Pseudomonas aeruginosa* (17), around one hundred Acrs have been discovered based on functional screening and bioinformatic analysis (18–20). Because Acr proteins frequently lack sequence homology and common structural motifs, they are classified based on the targeted CRISPR-Cas systems (16,18).

The AcrIF family inhibits the type I-F CRISPR-Cas system. Since the first AcrIF proteins (AcrIF1–5) were identified (17), 19 additional AcrIF proteins (AcrIF6–24) were discovered (21–23). The AcrIF family has also been a major focus of efforts to elucidate the structures and mechanisms of Acr proteins. These studies showed that the AcrIF family blocks type I-F activity in three distinct ways. The most common strategy is to block target DNA recognition by Cascade by direct binding of the Acr to Cascade component proteins. AcrIF1 (24), AcrIF2 (25), AcrIF4 (26), AcrIF6 (27), AcrIF7 (26), AcrIF8 (27), AcrIF10 (28) and AcrIF14 (26) use this mechanism to inhibit the type I-F CRISPR-Cas system. The second strategy is to inhibit Cas3 by direct interaction with the Acr. Masking the active site of Cas3 by AcrIF3 inhibits Cas3 interactions to target DNA and Cas3 bound by AcrIF3 fails to cleave target DNA (29). The final characterized inhibition strategy of the AcrIF family is enzymatic activity, represented by AcrIF11. AcrIF11 has an ADP-ribosyltransferase activity which mediates the ADP-ribosylation of the Cascade complex to prevent target DNA binding (30).

AcrIF24 is among the most recently identified members of the AcrIF family. Interestingly, genetic analysis suggested that AcrIF24 has dual function as an Acr and an anti-CRISPR-associated (Aca) protein (23). Aca proteins are transcriptional regulators which commonly inhibit the expression of *acr* genes by forming an *acr-aca* operon (31–34). To understand the molecular basis underlying the functional mechanisms of AcrIF24, we determined its crystal structure. Our structure complements and adds to an AcrIF24 structural study that was published while our manuscript was in preparation (35). Based on our structural, microbiological and biochemical studies, we demonstrate the working mechanism of AcrIF24 as both an Acr and an Aca protein.

## MATERIALS AND METHODS

### Cloning, overexpression, and purification of AcrIF24, Cascade, Cas2/3 used for structural and biochemical studies

Primer sequences used in this study are listed in Supplementary Table S1.

The full-length *acrIF24* gene (encoding residues 1–228) from a *Pseudomonas aeruginosa* prophage (GenBank accession: WP_043084540) (23) was synthesized by Bionics (Daejeon, Republic of Korea) and cloned into a pET21a plasmid vector (Novagen, Madison, WI, USA). The NdeI and XhoI restriction sites were used for cloning. The resulting recombinant construct was transformed into *Escherichia coli* BL21(DE3) competent cells that were further cultured at 37°C in 1 l of lysogeny broth (LB) containing 50 μg/ml kanamycin. When the optical density at 600 nm (OD_600_) reached around 0.8, the temperature was adjusted to 20°C, and 0.5 mM isopropyl β-d-1-thiogalactopyranoside (IPTG) was added for induction of *acrIF24* expression. The induced cells were further cultured for 20 h in a shaking incubator at 20°C.

The cultured cells were harvested by centrifugation at 2000 × *g* for 15 min at 4°C, resuspended in 25 ml lysis buffer (20 mM Tris–HCl pH 8.0 and 500 mM NaCl), and lysed by ultrasonication. The cell lysate and supernatant were separated by centrifugation at 10 000 × *g* for 30 min at 4°C. The collected supernatant was mixed with Ni-nitrilotriacetic acid (NTA) affinity resins for 3 h, and the mixture was loaded onto a gravity-flow column (Bio-Rad, Hercules, CA, USA). The resin in the column was washed with 50 ml of lysis buffer to wash out unbound proteins. After washing, 3 ml elution buffer (20 mM Tris–HCl pH 8.0, 500 mM NaCl and 250 mM imidazole) was added to the column to elute the Ni-NTA bound target protein from the resin.

Eluted AcrIF24 was concentrated to 30 mg/ml and applied onto a Superdex 200 10/300 GL column (GE Healthcare, Waukesha, WI, USA) connected to an ÄKTA Explorer system (GE Healthcare), which had been pre-equilibrated with SEC buffer (20 mM Tris–HCl pH 8.0 and 150 mM NaCl) for polishing the protein sample by size-exclusion chromatography (SEC). The eluted peak fractions from SEC containing AcrIF24 were collected, pooled, and concentrated to 12 mg/ml for crystallization. The purity of the protein was visually assessed using sodium dodecyl sulfate-polyacrylamide gel electrophoresis (SDS-PAGE).

For obtaining type I-F Cascade complex, the plasmids pCsy_complex (#89232) and pCRISPR_DMS3g24 (#89244) were purchased from Addgene (36,37) and co-transformed into *E. coli* BL21 (DE3) cells. The Cascade complex was purified using the same method used for AcrIF24 purification. To express and purify individual subunits of Cascade, the full-length *cas5, cas6, cas7* and *cas8* genes were obtained by PCR using the pCsy_Complex plasmid as template DNA. Each obtained gene was cloned into a pET21a plasmid vector using NdeI and XhoI restriction sites. The purification methods used for Cas5, Cas6, Cas7 and Cas8 were the same as for purification of AcrIF24.

The sequence encoding the AcrIF24 head domain deletion mutant was synthesized by Bionics (Daejeon, Republic of Korea), cloned into a pET21a plasmid vector (Novagen) and purified using the same method as for AcrIF24.

Cas2/3 protein was prepared by a previously described purification method using the Cas2/3 expression construct obtained from the laboratory of Yue Feng (35)

### Crystallization and X-ray diffraction data collection

The hanging drop vapor diffusion method was used for the crystallization of AcrIF24. Crystal plates were incubated at 20°C. Initial crystals were obtained by equilibrating a mixture containing 1 μl of protein solution (12 mg/ml protein in SEC buffer) and 1 μl of a reservoir solution containing 24% (w/v) polyethylene glycol 3350 (PEG 3350) and 0.4 M ammonium chloride (NH_4_Cl) against 500 μl of reservoir solution. The crystallization conditions were further optimized and the best crystals were obtained by adding 50 mM sodium fluoride (NaF), after which crystals appeared within 38 days. A single crystal was selected and soaked in reservoir solution supplemented with 40% (v/v) glycerol for cryo-protection. X-ray diffraction data were collected at −178°C on the beamline BL-5C at the Pohang Accelerator Laboratory (Pohang, Korea). Data was processed using HKL2000 software (38).

### Structure determination and refinement

The AcrIF24 structure was determined using the molecular replacement phasing method which was performed by the PHASER program in the PHENIX package (39). The predicted structural model generated by AlphaFold2 was used as a search model (40). The initial model was built automatically using AutoBuild from the PHENIX package, and further model building with refinement was performed using Coot (41) and phenix.refine (42). The structure quality and stereochemistry were validated using MolProbity (43). All structural figures were generated using PyMOL (44).

### Mutagenesis

Site-directed mutagenesis was performed using a Quick-change kit (Stratagene, San Diego, CA, USA) according to the manufacturer's protocols. Mutations were then confirmed by sequencing. Primer sequences used for mutagenesis are listed in Supplementary Table S1. Mutant proteins were prepared using the same method described for wildtype protein purification above.

### Multi-angle light scattering analysis (MALS)

The absolute molecular weight of AcrIF24 in solution was measured using SEC-coupled multi-angle light scattering (SEC-MALS). The protein solution was loaded onto a Superdex 200 Increase 10/300 GL 24 ml column (GE Healthcare) pre-equilibrated with SEC buffer. The flow rate of the buffer was controlled to 0.4 ml/min, and SEC-MALS was performed at 20°C. A DAWN-TREOS MALS detector was connected to an ÄKTA Explorer system. The molecular weight of bovine serum albumin was measured as a reference value. Data were processed and assessed using ASTRA software.

### Sequence alignment

The amino acid sequences of AcrIF24 across different species were analyzed using Clustal Omega (45).

### Identification of the AcrIF24 binding site

Promoter elements upstream of *acrIF23* (which forms an operon with *acrIF24*) were identified using BPROM (46) and by manual curation compared to established consensus sequences. Inverted repeat sequences were identified using the Repeat Finder plugin of Geneious Prime Version 2022 1.1 (https://www.geneious.com).

### Construction of plasmids for reporter assays and phage infection assays

The gBlock PF5881 was used as a template for amplification of the *acrIF23-acrIF24* promoter using primers PF138 and PF139, and of the *acrIF24* gene using PF209 and PF210. Oligonucleotide sequences used in this study are listed in Supplementary Table S1. The promoter PCR product was cut with SpeI and NsiI and inserted into pPF1439, a template plasmid for *eyfp* reporter studies (47), digested with the same enzymes, yielding pPF2963. The gene PCR product was cut with SacI and SphI and inserted into pBAD30 digested with the same enzymes, yielding pPF2964. To generate expression constructs for AcrIF24 variants with single point mutations, PCRs of the entire pPF2964 plasmid were performed with the primer pairs PF6867 and PF6868 (W110K), PF6869 and PF6870 (K197W) or PF6871 and PF6872 (R207W). PCR products were gel-purified and digested with DpnI to degrade the template plasmid, then directly transformed into *E. coli* DH5α to generate the plasmids pPF3482, pPF3483 and pPF3484, respectively. To generate the construct for the AcrIF24 double-mutant (Y128K/Y217W), the gBlock PF6976 was inserted into pBAD30 via the SacI and SphI sites, yielding pPF3487. All new plasmid constructs were confirmed by Sanger sequencing.

### Reporter assays

Reporter assays were performed using the *P. carotovorum* derivative PCF425, which has a deletion of two restriction endonuclease genes (48). Strains containing different combinations of the reporter plasmid with the *acrIF23-acrIF24* promoter (pPF2963) and the *acrIF24* expression plasmid (pPF2964 for the wild type or pPF3482, pPF3483, pPF3484 or pPF3487 for the different mutants) or the corresponding empty vectors (pPF1439 and pBAD30, respectively) were grown in LB containing 100 μg/ml ampicillin, 25 μg/ml chloramphenicol, 0.1% (w/v) arabinose and 100 μM IPTG at 1,200 rpm at 25°C. After 18 h of growth, fluorescence of plasmid-encoded mCherry and eYFP was measured by flow cytometry using a BD LSRFortessa cell analyzer. Cells were first gated based on forward and side scatter and cells positive for mCherry fluorescence were detected using a 610/20-nm bandpass filter (detector gain 606 V) and further analysed for eYFP fluorescence using a 530/30-nm bandpass filter (detector gain 600 V). Results for a strain carrying two empty vectors (pPF1439 and pBAD30) were subtracted from all other results to account for background fluorescence.

### Generation of a *P. carotovorum* strain with CRISPR resistance to phage ZF40

Examination of the *in vivo* activity of AcrIF24 against a phage-targeting CRISPR-Cas system first required creating a resistant host with a phage-targeting spacer in a type I-F CRISPR array. To generate a vector to promote acquisition of spacers targeting phage ZF40 in the *P. carotovorum* derivative PCF425, a 2.4kb-fragment of an uncharacterized ZF40 gene (locus tag F396_gp65) was first amplified by PCR using the primers PF2910 and PF2911 with a ZF40 lysate as the template. The resulting product was inserted into pPF1123 (49) using the restriction enzymes KpnI and SacI, yielding the plasmid pPF1526. Next, the oligonucleotides PF2914 and PF2958 were annealed. The annealing product contains a protospacer targeted by the *P. carotovorum* RC5297 type I-F CRISPR–Cas system combined with a non-consensus protospacer-adjacent motif (5′-TG-3′) previously shown to elicit priming in a related strain (50). This annealing product, which has SphI and SpeI restriction overhangs, was inserted into pPF1526 cut with the same enzymes, resulting in the spacer acquisition plasmid pPF1527.

For spacer acquisition via priming, strain PCF425 was transformed with pPF1527 by electroporation. Plasmid uptake was expected to trigger spacer acquisition due to the presence of the priming protospacer on pPF1527, with acquisition potentially occurring from the plasmid-born ZF40 gene fragment. Resulting transformants were grown for 24 h at 30°C in LB medium without antibiotic selection to allow plasmid loss and then plated on LB-agar plates containing 100 μM IPTG to allow selection against colonies producing mCherry, which is encoded on pPF1527. Of mCherry-negative colonies, arrays of the type I-F CRISPR-Cas system were screened for spacer acquisition using PF2969 and PF2970 (array 1) or PF2971 and PF2972 (array 2). Expanded arrays were sequenced to identify the origin of the newly acquired spacers. One of the resulting strains, named PCF835, which acquired one spacer targeting phage ZF40, was selected for further experiments.

### Phage-based AcrIF24 activity assay

To test the activity of AcrIF24 variants against the native type I-F CRISPR-Cas system of *P. carotovorum* RC5297, pBAD30 or a derived construct for production of an AcrIF24 variant (pPF2964 for the wild type or pPF3482, pPF3483, pPF3484 or pPF3487 for the different mutants) was electroporated into strain PCF425 (without CRISPR immunity against phage ZF40) or PCF835 (with type I-F CRISPR immunity against phage ZF40). Each strain was grown overnight in LB medium containing 0.2% (w/v) arabinose for induction. From these cultures, 100 μl were added to top agar and poured on LB-agar plates containing 0.2% (w/v) arabinose. After solidification, 5 μl spots of a tenfold serial dilution of a ZF40 variant with its native *acrIF8–aca2* operon deleted were placed on the top agar. Plaque formation was examined after 16 h of incubation at 25°C.

### Electrophoretic mobility shift assay with polyacrylamide gel (EMSA-P)

Varying concentrations of purified wildtype or mutant AcrIF24 were pre-incubated with 20 ng of annealed long inverted repeat DNA (IR-L) or 800 ng of annealed short inverted repeat DNA (IR-S) in binding buffer (10 mM HEPES pH 7.5, 1 mM MgCl_2_ 20 mM KCl, 1 mM tris(2-carboxyethyl)phosphine (TCEP), and 5% (v/v) glycerol in a final volume of 20 μl) for 30 min on ice. Prepared samples were then separated by gel electrophoresis at 100 V on a 10% native 0.5× TBE (Tris–borate–EDTA) polyacrylamide gel. After electrophoresis, gels were stained with SYBR Gold (Invitrogen, Waltham, MA, USA) and visualized according to the manufacturer's instructions. Annealed DNA oligos, IR-L and IR-S, were generated by mixing complementary oligonucleotides synthesized by Bionix (Seoul, Republic of Korea) in a 1:1 molar ratio in annealing buffer (10 mM Tris pH 7.5, 50 mM NaCl, and 1 mM EDTA), heating to 100°C for 3 min, and cooling to 25°C for 1 h.

### Electrophoretic mobility shift assay with agarose gel (EMSA-A)

Purified AcrIF24 (wildtype and various mutants) at a concentration of 20 μM were pre-incubated with 1.5 μg annealed oligo DNA (IR-L or IR-S) at 4°C for 60 min in a final volume of 20 μl SEC buffer. Agarose gels (6%) were prepared with agarose LE powder (Gold Biotechnology) using 0.5× TB buffer. Prepared agarose gel was run on a Mupid-2 plus electrophoresis kit (Advance, Japan) in 0.5× TB buffer for 30 min at 100 V.

### Native-PAGE

Protein-DNA complex formation between AcrIF24 (wildtype or mutants) and annealed oligo DNA (IR-L and IR-S) was evaluated via native (non-denaturing) PAGE with 8∼25% acrylamide gels. Coomassie Brilliant Blue was used for staining and detection of shifted bands. DNA and protein were used at concentrations of 10 and 20 μM, respectively.

### Size-exclusion chromatography assay for complex formation

SEC was performed to analyze complex formation between AcrIF24 and type I-F Cascade. AcrIF24 was mixed with type I-F Cascade or each individual protein component of Cascade, incubated for 30 min at 25°C, and applied to a size-exclusion column (Superdex 200 HR 10/30, GE healthcare), which was pre-equilibrated with SEC buffer. The peak fractions were collected and subjected to SDS-PAGE. Coomassie Brilliant Blue was used for staining and analyzing the pattern of co-migrated bands.

### *In vitro* anti-CRISPR activity assay

To test the anti-CRISPR activity of wildtype AcrIF24 and its mutants (W110K, ΔHead, K197Y, R207W, and Y128K/Y217W), reactions were performed in a 10 μl buffer system containing 0.64 μM Cascade complex, 0.16 μM Cas2/3, 0.04 μM dsDNA, and 100–1000 nM AcrIF24 or its mutant proteins. First, we incubated AcrIF24 or its mutants with the type I-F Cascade complex at 37°C in reaction buffer (20 mM HEPES pH 7.5, 100 mM KCl, 5% (v/v) glycerol, and 1 mM DTT) for 30 min. Then we added target DNA to a final concentration 0.04 μM and incubated at 37°C for 30 min. Cas2/3 was further added along with reaction buffer (5 mM MgCl_2_, 75 μM NiSO_4_, 5 mM CaCl_2_ and 1 mM ATP) and the reaction was performed at 37°C for 1 h. We quenched the reaction by adding proteinase K and incubating for an additional 10 min at room temperature. The reaction products were separated by electrophoresis on 10% polyacrylamide gels and visualized by staining with SYBR GOLD.

## RESULTS

### AcrIF24 from a *P. aeruginosa* prophage has three distinct domains

**Figure 1.**
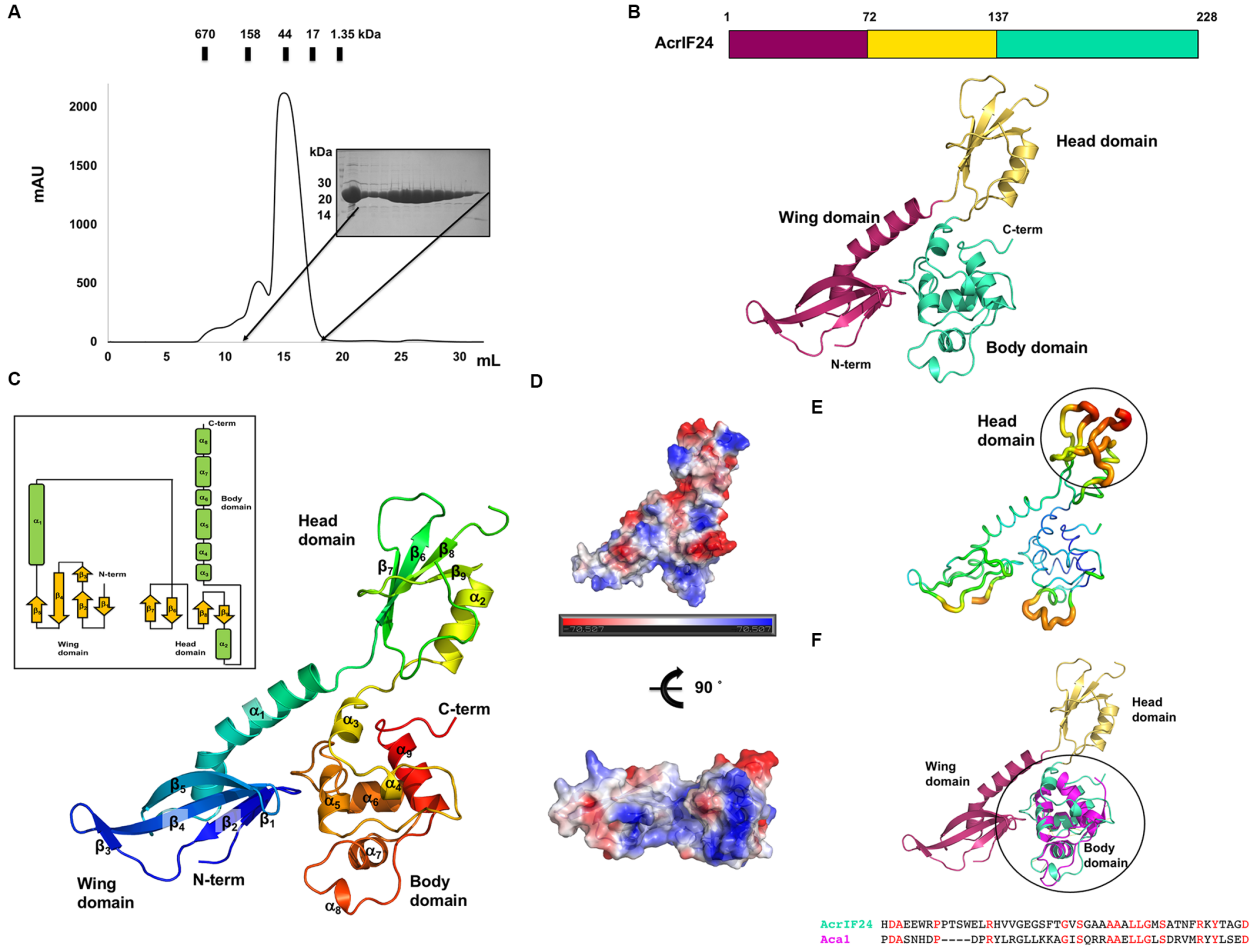
The crystal structure of AcrIF24 reveals a novel three-domain arrangement. (**A**) Size-exclusion chromatography (SEC) profile of AcrIF24. An SDS-PAGE gel loaded with the peak fractions is provided to the right of the main peak. The corresponding fractions from SEC loaded onto SDS-PAGE are indicated by black arrows. (**B**) The overall structure of AcrIF24 has three distinct domains (wing, head and body). (**C**) Cartoon representation of AcrIF24. The color of the chain from the N- to the C-termini gradually moves through the spectrum from blue to red. The nine α-helices and nine β-sheets are labeled α_1_–α_9_ and β_1_–β_9_, respectively. Topology representation of AcrIF24 is provided in the inset. (**D**) Surface electrostatic potential of AcrIF24. The respective surface electrostatic distributions are represented by a scale ranging from −7.0 kT/e (red) to 7.0 kT/e (blue). (**E**) *B*-factor distribution in the structure of AcrIF24. The structure is presented in a putty representation. Rainbow colors from red to violet with increasing *B*-factor values were used for *B*-factor visualization. The region with the highest B-factor, corresponding to most of the head domain, is indicated by a black circle. (**F**) Structural superimposition of Aca1 with AcrIF24. The superposed reqion is indicated by a black circle. The magenta cartoon model represents the structure of Aca1.

To understand the molecular basis underlying AcrIF24 anti-CRISPR function, AcrIF24 was overexpressed in *E. coli* and purified using two-step chromatography, affinity chromatography and size-exclusion chromatography (SEC). During SEC, the protein eluted at bigger than ∼44 kDa from a Superdex 200 gel-filtration column, indicating that AcrIF24 (∼26 kDa for monomer) may exist as a dimer in solution (Figure 1A). The purified AcrIF24 protein sample was successfully crystallized and diffracted to 2.5 Å.

Due to the absence of structural homologues in the PDB database, we were unable to solve the phasing problem by molecular replacement (MR) at the initial stage of structure determination. However, the phasing problem was solved by molecular replacement using a structural model predicted by alphafold2 (40). The final structural model of AcrIF24 was refined to *R*_work_ = 21.66% and *R*_free_ = 28.58%. The diffraction data and refinement statistics are summarized in Table 1. The crystal belongs to space group *P6_5_22* with one molecule present in the asymmetric unit. The final structural model contains the complete AcrIF24 sequence (residues M1 to S228). The overall shape of the AcrIF24 structure resembled a bird and was composed of three distinct domains. Based on the domain locations in the bird shape, we named them head, wing and body domain (Figure 1B). The wing domain was formed by five β-sheets (β_1_–β_5_) and one long α-helix (α_1_) at the N-terminal part of AcrIF24, while the body domain was composed of six α-helixes (α_3_-α_8_) forming a helical bundle fold at the C-terminus of AcrIF24 (Figure 1C). The head domain was formed by four β-sheets (β_6_–β_9_) and one α-helix (α_2_) was localized between the wing and body domains (Figure 1C).

**Table 1.** Data collection and refinement statistics

Data collection	
Space group	*P 6_5_22*
Unit cell parameter *a*, *b*, *c* (Å)	
*a*, *b*, *c* (Å)	62.17, 62.17, 229.1
α, β, γ (°)	90, 90, 120
Resolution range (Å)	27.97–2.53
Total reflections	356 590
Unique reflections	9494
Multiplicity	37.6 (40.2)
Completeness (%)^a^	99.86 (100.00)
Mean *I*/σ(*I*)^a^	19.75 (3.12)
*R*_merge_ (%)^a^^,^^b^	19.25 (26.95)
Wilson *B*-factor (Å^2^)	58.23
**Refinement**	
Resolution range (Å)	27.97–2.53
Reflections	9,493
*R*_work_ (%)	21.66
*R*_free_ (%)	28.56
No. of molecules in the asymmetric unit	1
No. of non-hydrogen atoms	1796
Macromolecules	1737
Solvent	59
Average *B*-factor values (Å^2^)	59.82
Macromolecules	59.87
Solvent	50.44
Ramachandran plot:	
favored/allowed/outliers (%)	98.64/1.36/0.00
Rotamer outliers (%)	0.00
Clashscore	4.36
RMSD bonds (Å)/angles (°)	0.007/0.951

^a^Values for the outermost resolution shell in parentheses.

^b^ *R*_merge_ = Σ_h_ Σ_i_ |*I*(*h*)_i_ − <*I*(*h*)>|/ Σ*_h_* Σ*_i_* I(*h*)*_i_*, where *I*(*h*) is the observed intensity of reflection *h*, and < *I*(*h*)> is the average intensity obtained from multiple measurements.

Because the electrostatic surface features are sometimes important for predicting the function of a protein, we analyzed the electrostatic surface features of AcrIF24. This analysis showed that AcrIF24 contained a highly positively charged cleft in the bottom part of the body domain, although negatively and positively charged surfaces were evenly dispersed in the AcrIF24 structure (Figure 1D). *B*-factor analysis showed that most of the head domain had relatively higher *B*-factors (average 88.25 Å^2^), indicating that the head domain might be flexible (Figure 1E). The α_8_ helix and connecting loop at the body domain also had relatively higher *B*-factors (average 62.37 Å^2^). Because flexible protein features can become rigid upon interaction with a specific binding partner, the flexible head domain might be critical for the protein interactions for the proper function of AcrIF24.

To investigate the structural novelty of AcrIF24, structural homologues were searched using the DALI server (51). The closest related structure picked by this server was Aca1 (52), having a *Z*-score of 6.9 and 2.5 Å root mean square deviation (RMSD) when superimposing 68 amino acids among 73 total amino acids of Aca1 with 72 amino acids among 228 total amino acids of AcrIF24 (Table 2). The structure of Aca1 was only superposed with the body domain of AcrIF24 (Figure 1F). The sequence identity of Aca1 with the body domain of AcrIF24 was 22%. This search indicated that the structures of the wing and head domains of AcrIF24 are novel without significant similarity to previously described structures. Although the overall structure of the body domain of AcrIF24 is similar to the entire structure of Aca family, structural superposition indicated that the two structures are not identical, having a high RMSD value (2.5 Å).

**Table 2.** Structural similarity search using DALI (51)

Proteins (accession numbers)	*Z*-score	RMSD (Å)	Identity (%)
Aca1 (7C0H)	6.9	2.5 (68/73)	22
MMOX1 (6D7K)	6.2	2.5 (61/64)	8
SO3848 (2OX6)	6.0	1.6 (55/162)	16
Aca2 (7B5J)	5.8	1.9 (53/115)	17
Putative DNA binding protein (3BS3)	5.8	2.1 (54/62)	13
CLGR (3F51)	5.7	1.6 (54/94)	17
CSP231I (3LFP)	5.5	1.6 (55/96)	16
MPnS (6B9T)	5.4	1.7 (54/447)	19
DDROC (6RNZ)	5.4	1.6 (53/66)	15
HigA2 (5J9I)	5.3	2.7 (61/69)	15
ACID epoxidase (1ZZC-A)	5.1	1.9 (54/179)	17

### AcrIF24 forms a dimer via the head and body domains

**Figure 2.**
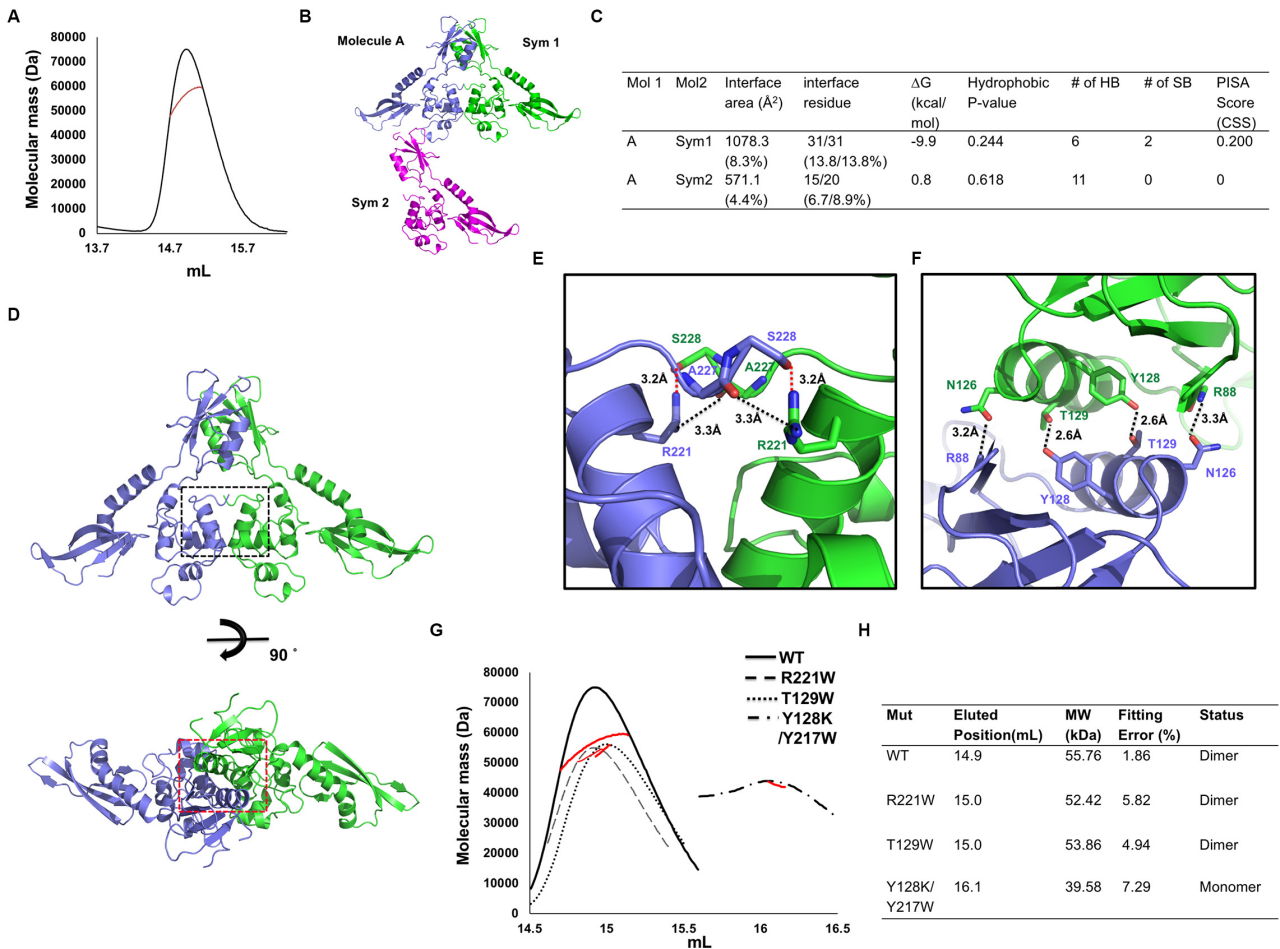
Dimeric structure of AcrIF24. (**A**) Multi-angle light scattering (MALS) profile of AcrIF24. The experimental MALS data (red line) are plotted as SEC elution volume (x-axis) versus absolute molecular mass (y-axis) distributions on the SEC chromatogram (black) at 280 nm. (**B**) Crystallographic packing symmetry analysis. A single molecule found in the asymmetric unit is indicated by the purple-colored ribbon structure and the two symmetry molecules found by packing analysis are indicated by green-colored (Sym1) and magenta-colored (Sym2) ribbon structures. (**C**) Table summarizing the interaction details of the two types of putative interfaces analyzed by the PISA server. (**D**) Putative dimeric structure of AcrIF24 generated and analyzed by crystal packing and PISA server. The regions of PPI magnified and presented in (E and F) are indicated by a black-dashed square for region 1 (body domain-mediated PPI) and red-dashed square for region 2 (head domain-mediated PPI). (E and F) Close-up views of two different PPIs, region 1 (**E**) and region 2 (**F**), in the dimeric structure of AcrIF24. The red-dashed and black-dashed lines indicate salt bridges and hydrogen bonds, respectively. (**G**) Validation of the PPI via mutagenesis. SEC-MALS profiles comparing the position of eluted peaks of various mutants with wildtype. The red line indicates the experimental molecular mass measured by MALS. (**H**) Table summarizing the result of SEC-MALS. Mut and MW indicate mutant and molecular weight, respectively. Fitting error indicates the MALS fitting error.

Although various Acrs inhibit CRISPR-Cas activity in monomeric form, previous studies have shown that the dimeric form of Acrs is often critical for their activity (29,53,54). Similarly, some Aca proteins have been shown to function as dimers (31,33,55). Given the possible dimeric form of AcrIF24 as judged by our SEC experiment, we used multi-angle light scattering (MALS) to confirm the stoichiometry by determining the absolute molecular mass of AcrIF24 in solution. MALS showed that the experimental molecular mass was 55.76 kDa (1.86% fitting error) with 1.002 polydispersity (Figure 2A). Because the theoretically calculated molecular weight of monomeric AcrIF24 with the C-terminal histidine tag was 26.03 kDa, the molecular mass analyzed by MALS likely corresponds to dimeric AcrIF24. Based on these SEC and MALS data, we concluded that AcrIF24 forms a homo-dimer in solution.

Crystallographic packing analysis showed that two types of putative dimers were detected: a MolA/Sym1 dimer or a MolA/Sym2 dimer. The MolA/Sym1 dimer was constructed via head and body domains from each molecule, while the MolA/Sym2 dimer was formed via the wing and body domains from one molecule and the head domain of another molecule (Figure 2B). To find a symmetric molecule that forms a dimer with monomeric molecule A found in the crystallographic asymmetric unit, the protein-protein interactions (PPI) in both the MolA/Sym1 dimer and the MolA/Sym2 dimer were further analyzed using the PDBePISA PPI-calculating server (Figure 2C) (56). PPI analysis showed that the complex formation significance score (CSS) of the MolA/Sym1 dimer was 0.2 (the score ranges from 0 to 1 as the relevance of the interface to complex formation increases), while that of the MolA/Sym2 dimer was 0, indicating that the MolA/Sym1 dimer might be the biologically relevant form. A total of 62 residues (31 from each molecule) were involved in the formation of PPI of the MolA/Sym1 dimer, whose total surface buried an area of 1078.3 Å^2^, representing 8.3% of the total surface area (Figure 2C and D). Meanwhile, 15 residues from MolA and 20 residues from Sym2 were involved in the formation of the MolA/Sym2 PPI, whose total surface buried an area of 571.1 Å^2^, representing 4.4% of the total surface (Figure 2C). The main forces used for the formation of the MolA/Sym1 dimer PPI were six hydrogen bonds (H-bonds) and two salt bridges, which were generated at two distinct regions, one in the body domains and another in the head domains. For maintaining the head domain-mediated PPI, salt bridges were formed in between S228 and R221 of each molecule and H-bonds were formed between residues A227 and R221 of each molecule (Figure 2D and E). For maintaining the body domain-mediated PPI, extensive H-bonds were generated by R88, Y128, T129, and N126 of each molecule (Figure 2D and F). In contrast to the MolA/Sym1 dimer, the MolA/Sym2 dimer was an asymmetric dimer that might be unlikely to form in the cellular environment. Therefore, we propose that AcrIF24 naturally occurs as a MolA/Sym1 dimer.

To confirm our hypothesis that the MolA/Sym1 dimer might be a favoured dimer model, we generated three MolA/Sym1 PPI disruption mutants, R221W (head domain-mediated PPI disrupting mutant), T129W (wing domain-mediated PPI disrupting mutant), and a Y128K/Y217W double mutant (both PPI disrupting mutant) and performed SEC-MALS with those mutants. This experiment clearly showed that the Y128K/Y217W double mutant produced a peak at a delayed position during SEC compared with that of the wildtype. The Y128K/Y217W double mutant produced a smaller-sized particle, which is likely a monomer of AcrIF24, indicating that MolA/Sym1 represents the true dimeric state (Figure 2G and H).

### AcrIF24 has Aca activity and represses the*acrIF23-acrIF24* operon

**Figure 3.**
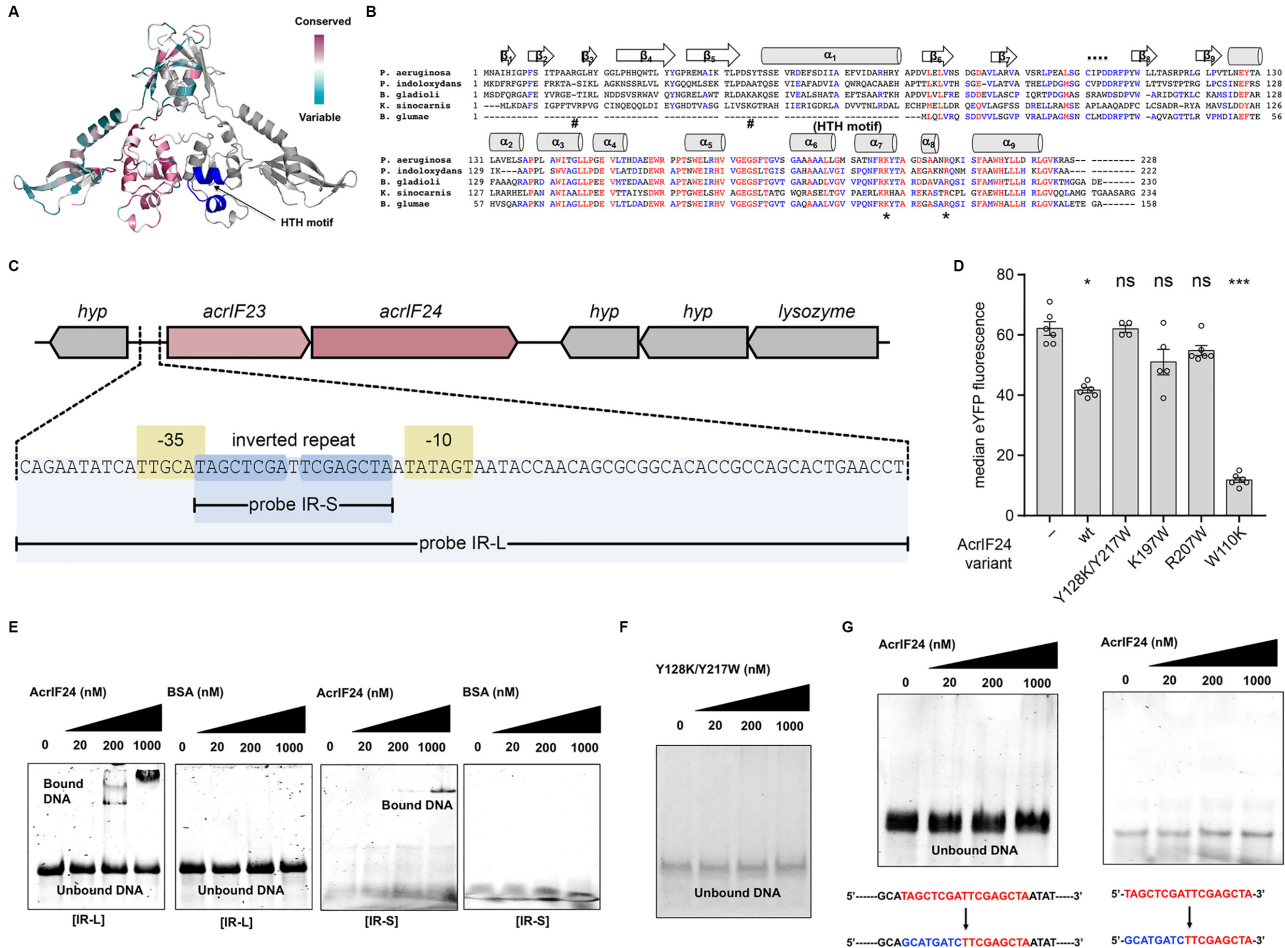
AcrIF24 possesses Aca function by binding an IR and repressing *acrIF23-acrIF24* promoter expression. (**A**) Cartoon representation of dimeric AcrIF24 colored according to the degree of amino-acid sequence conservation across different species as analyzed by the ConSurf server. (**B**) Sequence alignment of AcrIF24 between different species. Mostly conserved and partially conserved residues are colored in red and blue, respectively. The location of three helices α_6_−α_8_ containing the putative HTH motif are shown above the corresponding sequence. * indicates conserved residues that were involved in the DNA recognition. ^#^ indicates mutated residues in the wing domain that were predicted as DNA binding residues. (**C**) Genomic context of the *P. aeruginosa acrIF23-acrIF24* operon, with the predicted promoter enlarged. Regulatory elements (–35 and –10 regions) are highlighted in yellow, the inverted repeat is shown by blue arrows. The IR-S and IR-L probes used for EMSAs in (E) and (F) are indicated underneath. (**D**) Activity of an *acrIF23-acrIF24* promoter reporter in *P. carotovorum* in the presence and absence of AcrIF24 (wildtype and the indicated variants), determined as the median eYFP fluorescence by flow cytometry. Data are presented as the mean ± standard error, with individual replicates represented by dots; statistical significance was assessed by a Kruskal-Wallis test and Dunn's multiple comparisons test against the -AcrIF24 control (ns *P*≥ 0.05, * *P*< 0.05, *** *P*< 0.001). (**E**) Representative EMSA with AcrIF24 using IR-L and IR-S as a substrate. Purified AcrIF24 at the indicated concentrations was mixed with substrate DNA. Non-denaturing acrylamide gels stained with SYBR Gold are shown. (**F**) EMSA with dimer-disrupted mutant of AcrIF24 (Y128K/Y217W) using IR-L as a substrate. Non-denaturing acrylamide gels stained with SYBR Gold are shown (**G**) EMSA with AcrIF24 using half-site IR mutants of IR-L and IR-S as a substrate. Substituted bases are indicated in blue. Non-denaturing acrylamide gels stained with SYBR Gold are shown.

The dimeric nature of AcrIF24, the presence of a predicted helix-turn-helix (HTH) DNA binding motif similar to some known Aca proteins (23), and the high structural similarity of the body domain of AcrIF24 with Aca1 analysed by DALI search followed by structural superimposition (Table 2 and Figure 1F), led us to hypothesize that AcrIF24 functions analogously to an Aca protein by binding DNA and altering gene expression. Analysis of AcrIF24 using ConSurf demonstrated that the base of the body domain was highly conserved and contained the putative HTH motif within the C-terminus of the protein (Figure 3A), spanning helices α_6_–α_8_ (Figure 3B). Although the amino acid sequences of the head and body domains were highly conserved, the N-terminal wing domain was not conserved. Because the putative HTH motif was found in the body domain, AcrIF24 might use the body domain to recognise the specific promoter sequence for the Aca activity.

The dimeric nature of AcrIF24 suggested that it would likely recognise an inverted repeat (IR) sequence to control *acr* expression. In the *P. aeruginosa* prophage, *acrIF24* is present as the second gene in an operon also including *acrIF23*. Therefore, we predicted that the entire *acrIF23-acrIF24* operon would be regulated by AcrIF24. We examined the intergenic region upstream of *acrIF23* for a predicted promoter using BPROM and manual curation, which revealed –35 and –10 regions consistent with a strong promoter (TTGCAT-N17-TATAGT) (Figure 3C). Positioned almost perfectly between the -35 and -10 elements of this promoter was an IR (TAGCTCGATTCGAGCTA) with two perfect 8 bp half-sites (underlined) separated by 1 bp (Figure 3C).

To test whether AcrIF24 functions to regulate the *acrIF23-acrIF24* operon, we made a transcriptional fusion of the *acr* operon promoter to *eyfp*. Expression of *acrIF24* led to a reduction in eYFP fluorescence as assessed by flow cytometry, indicating that AcrIF24 functions as an Aca to repress *acr* operon expression (Figure 3D). To examine if AcrIF24 binds directly to the *acrIF23-acrIF24* promoter region, we performed electrophoretic mobility shift assays (EMSA) with purified AcrIF24. AcrIF24 bound in a concentration-dependent manner to a long DNA fragment [IR-L] that contained the promoter and the IR sequence, whereas BSA did not bind this DNA (Figure 3E). The IR sequence was sufficient for AcrIF24 recognition, since the protein also bound to a minimal 17 bp dsDNA fragment [IR-S] that solely contained the IR (Figure 3E). Since the dimer-disrupted mutant of AcrIF24 (Y128K/Y217W double mutant) lost its ability to form a complex with IR-L, we concluded that dimerization of AcrIF24 was critical for the recognition of IR sequence in the promoter (Figure 3F). Indeed, this mutant was unable to repress the promoter in our reporter assay (Figure 3D). The complete IR was necessary for AcrIF24 binding, because mutation of one half of the IR in either the long [IR-L] or short [IR-S] DNA fragments abrogated interactions (Figure 3G). In summary, AcrIF24 contains a C-terminal HTH motif and recognizes and binds an IR to repress *acrIF23-acrIF24* operon expression.

### The body domain of AcrIF24 is critical for promoter binding

**Figure 4.**
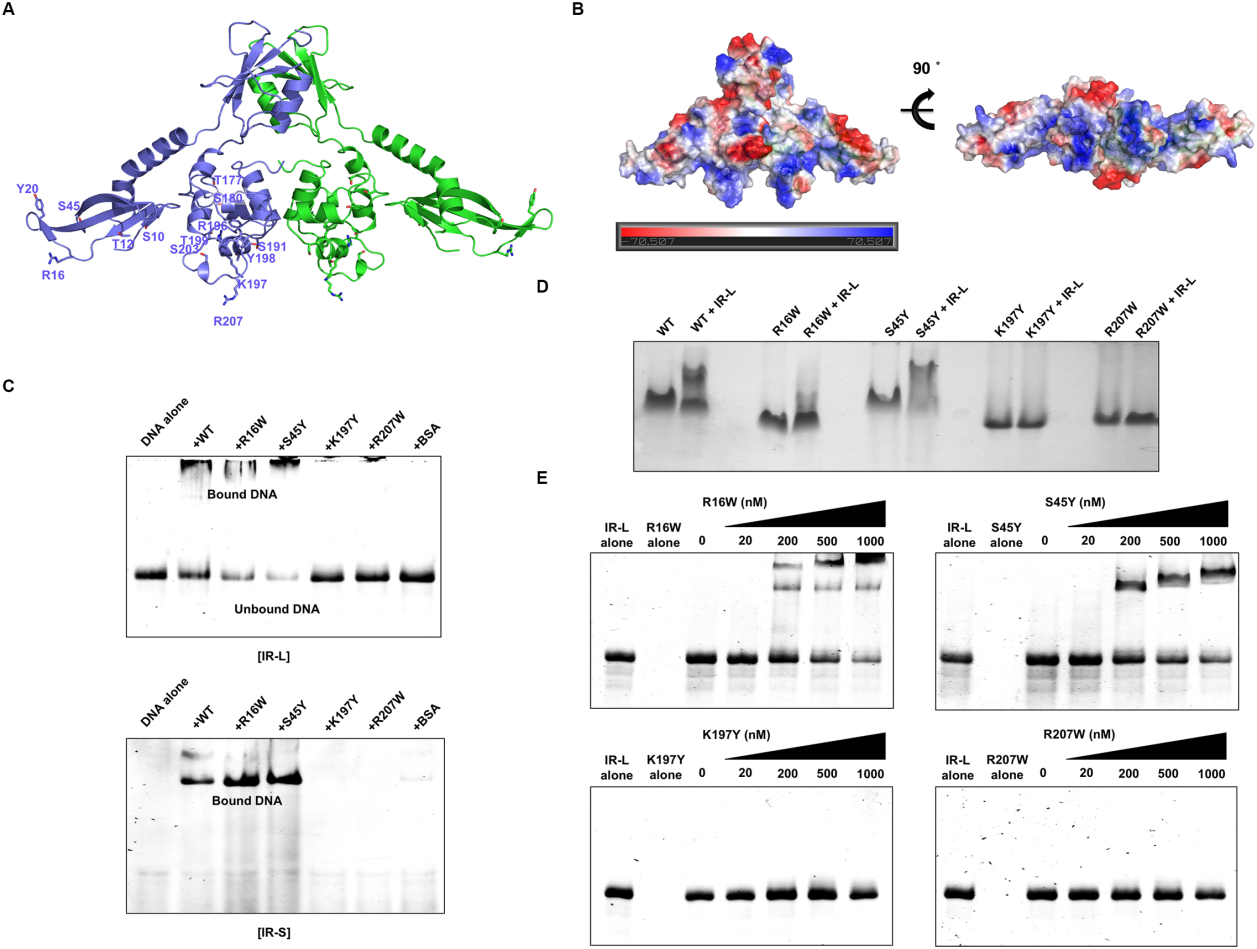
The HTH body domain of AcrIF24 is involved in DNA binding. (**A**) Predicted DNA binding residues on AcrIF24. (**B**) Surface electrostatic features of dimeric AcrIF24. The scale bar ranges from −7.0 kT/e (red) to 7.0 kT/e (blue). (**C**) EMSA with the indicated AcrIF24 mutants using IR-L and IR-S as a substrate. Purified proteins were added at concentration of 1 μM. BSA was used as a negative control. Non-denaturing acrylamide gels stained with SYBR Gold are shown. (**D**) Native-PAGE with the wildtype and indicated mutants using IR-L as a substrate. DNA binding ability of wildtype and each mutant of AcrIF24 were analyzed by comparison of band-shift by adding DNA substrate (IR-L). (**E**) EMSA with the indicated AcrIF24 mutants using IR-L in a various different concentration of protein. Non-denaturing acrylamide gels stained with SYBR Gold are shown.

After establishing a regulatory function for AcrIF24, we next aimed to identify the protein regions involved in DNA binding. A helix-turn-helix (HTH) domain was previously predicted at the C-terminus of the protein (23) which in our structure forms the body domain. To obtain a more detailed view of the DNA-binding residues on AcrIF24, we first used the DNA-binding residues prediction server, DRNApred (57). According to this prediction server, residues S10, T12, R16, Y20 and S45 on the wing domain and residues T177, S180, T199, R196, S191, Y198, K197, S203 and R207 on the body domain were selected as tentative DNA-binding residues (Figure 4A). Electrostatic surface features of dimeric AcrIF24, showing a highly positively charged cleft in the bottom part of the wing and body domains (Figure 4B), supported the DRNApred predictions. Based on these observations and predictions, we speculated that AcrIF24 may use its body domain to recognize and bind to the specific IR sequence of DNA and that the wing domain may also be involved in DNA recognition.

To test our hypothesis, we performed mutagenesis studies. Among the predicted tentative DNA binding residues, the highly conserved K197 and R207 on the body domain were mutated to tyrosine and tryptophan, respectively, producing K197Y and R207W mutants (Figure 3B). These two body domain mutants were hypothesized to disrupt DNA binding. For making wing domain mutants that might disrupt DNA binding, residues R16 and S45 were selected and mutated to tryptophan and tyrosine, respectively, generating R16W and S45Y mutants. These two residues were not conserved across different species (Figure 3B). All mutant proteins were purified and tested for DNA binding.

While R16W and S45Y wing domain mutants were still able to bind to DNA, the K197Y and R207W body HTH motif mutations completely abrogated binding to long and short promoter sequences (IR-L and IR-S) (Figure 4C and Supplementary Figure S1). We validated these results using EMSA in agarose gel and native-PAGE by detecting shifted protein bands generated by DNA interaction. As expected, K197Y and R207W mutants were not able to produce shifted bands, while wildtype and S45Y produced distinct shifts (Figure 4D and Supplementary Figure S2). Because the IR-L/AcrIF24 complex sometimes stuck in the well at the high concentration of AcrIF24 provided for EMSA assay, we verified the specific interaction of DNA and AcrIF24 using the same EMSA assay in a protein concentration-dependent manner (Figure 4E). These experiments more clearly showed that K197Y and R207W mutants completely lost their IR-L binding ability and R16W mutants partially lost its DNA binding capability (Figure 4E). All these binding experiments indicated that residues K197 and R207 in the body domain are critical for DNA binding. Although R16W produced a shift, the amount shifted was reduced compared with wildtype AcrIF24, indicating that R16 on the wing domain influences DNA binding (Figure 4D and E). In agreement with these results, the K197W and R207W mutant proteins were unable to repress the *acrIF23-acrIF24* promoter in reporter assays (Figure 3D). In conclusion, the HTH body domain of AcrIF24 is involved in recognition of the IR in the *acrIF23-acrIF24* promoter and this interaction is essential for promoter repression.

### AcrIF24 binds directly to Cascade via Cas7

**Figure 5.**
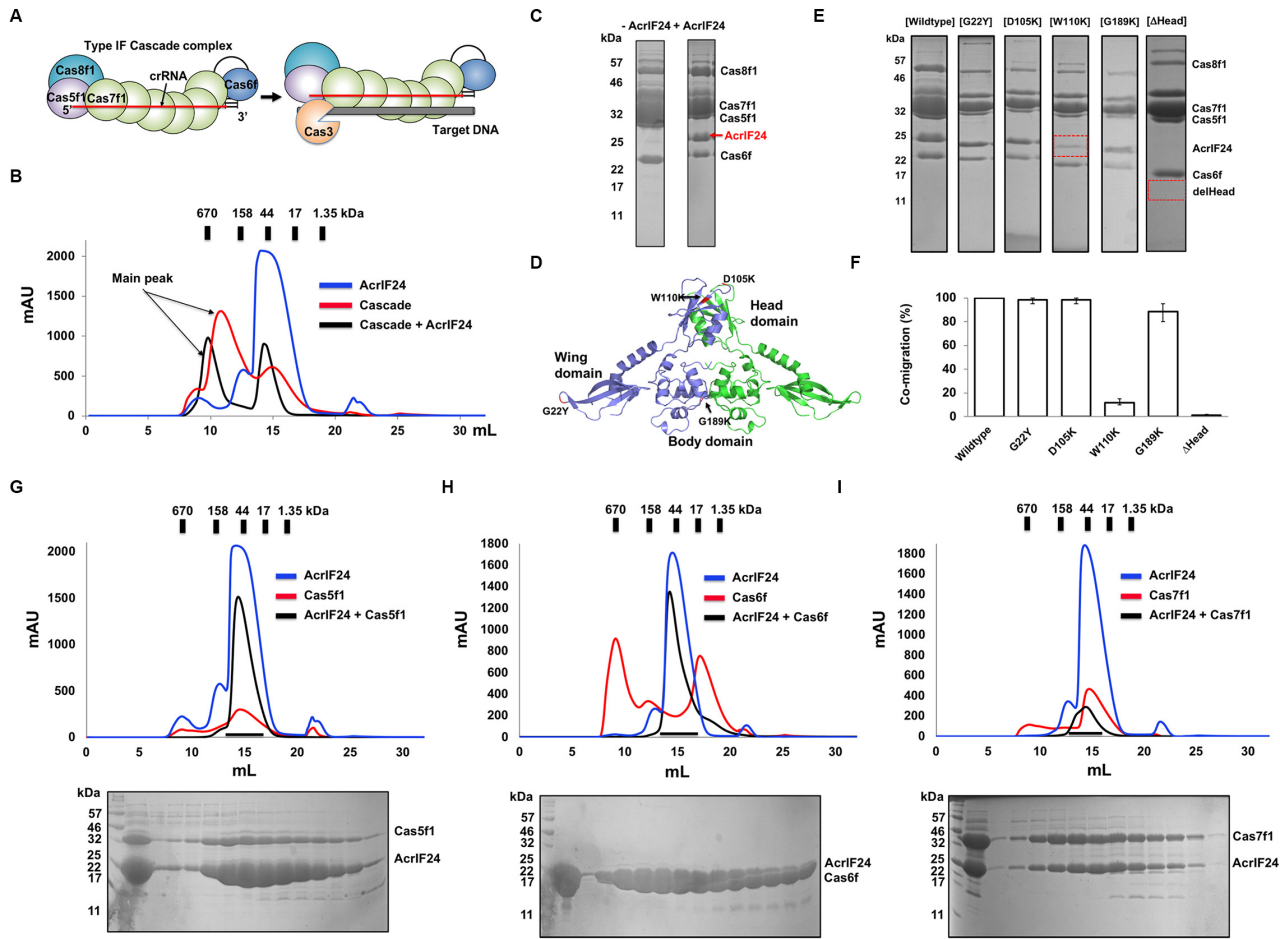
AcrIF24 directly binds type I-F Cascade via Cas7f1. (**A**) Schematic showing the composition and interference process by the type I-F Cascade complex. The crRNA processed and bound by Cas6f acts as a scaffold for type I-F Cascade assembly. (**B**) Interaction analysis between AcrIF24 and Cascade by SEC. SEC profiles produced by AcrIF24 (blue line), Cascade (red line), and the mixture of AcrIF24 and Cascade (black line) are shown. (**C**) SDS-PAGE gels produced by loading one of the main fractions from Cascade sample with (+AcrIF24) or without (–AcrIF24) providing AcrIF24. The position of each Cascade subunit on the gel is indicated. AcrIF24 co-migrated with Cascade is indicated by a red arrow. (**D**) Position of mutated residues marked on the dimeric structure of AcrIF24. Mutated residues G22Y (representative wing domain disruption mutant), D105K and W110K (representative head domain disruption mutants), and G189K (representative body domain disruption mutant) were indicated by red color. (**E**) Interaction analysis of Cascade with wildtype AcrIF24 and with various mutants of AcrIF24 by SEC followed by SDS-PAGE. SDS-PAGE gels produced by loading one of the main fractions from Cascade sample with various mutants of AcrIF24. The position of each Cascade subunit and AcrIF24 on the gel is indicated. The reduced co-purification of AcrIF24 (W110K) with Cascade is denoted on the gel by red dot-box. The tentative position of the ΔHead mutant that did not co-migrate with Cascade is also denoted in the gel by red dot-box. (**F**) Bar-chart showing the quantified intensity of the co-eluted AcrIF24 and various mutants on SDS-PAGE provided in (E). Data are presented as the mean ± standard deviation of three independent experiments. (**G–I**) Interaction analysis between AcrIF24 and each Cascade subunit by SEC. SEC profiles produced by AcrIF24 (blue line), each Cascade subunit (red line) of Cas5f1 (**G**), Cas6f (**H**) and Cas7f1 (**I**), and the mixture of AcrIF24 and each Cascade subunit (black line) are shown. SDS-PAGE gel produced by the mixture of AcrIF24 and each Cascade subunit is provided under the SEC profile. Loaded fractions for SDS-PAGE were indicated by a black bar.

To understand how AcrIF24 functions as an anti-CRISPR, we initially tested a direct interaction of AcrIF24 with the *P. aeruginosa* type I-F Cascade complex. This type I-F complex is composed of the Cas proteins Cas5f1, Cas6f, six copies of Cas7f1 and Cas8f1, and includes a single 60 nt crRNA (Figure 5A). First, SEC and SDS-PAGE were performed with Cascade in the absence of AcrIF24 to obtain a SEC profile and the location of each Cascade subunit on an SDS-PAGE gel. This analysis showed that the main peak fraction of the SEC profile, containing all the Cascade subunits, was produced at a position corresponding to an ∼400 kDa size particle eluted from the SEC column (Figure 5B and C and Supplementary Figure S3A and B). Since the typical mass of type-I-F Cascade is around 400 kDa, this SEC analysis indicated that the complex used in this study was successfully purified. In addition, each subunit of Cascade, Cas5f1 (36.2 kDa), Cas6f (21.4 kDa), Cas7f1 (39.7 kDa) and Cas8f1 (50.1 kDa), was detected at the expected position on the SDS-PAGE gel as well (Figure 5C and Supplementary Figure S3B).

To analyze a direct interaction of AcrIF24 with type I-F Cascade, purified AcrIF24 was mixed with purified Cascade complex, incubated and loaded onto the SEC column. This SEC experiment showed that the main peak of AcrIF24-Cascade eluted 1 ml earlier than that of Cascade lacking AcrIF24, suggesting that AcrIF24 and Cascade were interacting to form a larger complex (Figure 5B). This elution volume corresponds to a mass >670 kDa, suggesting that AcrIF24 interacts with Cascade and that this interaction leads to the aggregation of more than one Cascade complex with AcrIF24. Indeed, elution fractions from this major peak contained all Cascade subunits in addition to co-eluting AcrIF24 when visualized by SDS-PAGE (Figure 5C and Supplementary Figure S4A and B). These observations clearly showed that AcrIF24 directly interacted with Cascade.

To determine which region of AcrIF24 mediates binding to Cascade, we mutagenized AcrIF24. Because AcrIF24 was divided into the three distinct domains (wing, head, and body), we selected conserved and exposed surface residues from these domains and mutated them to residues that may disrupt the interaction with Cascade. G22Y and G189K mutants represented wing domain and body domain disruption mutants, respectively, while both D105K and W110K mutants represented head domain disruption mutants (Figure 5D). To test if the mutations affected interaction with Cascade, we performed SEC and SDS-PAGE. The main peak eluted at almost the same volume on the SEC profile for each mutant (Supplementary Figure S5A-E). However, co-migration of AcrIF24 W110K with Cascade was significantly reduced (Figure 5E and F, and Supplementary Figure S5D), indicating that this head domain disruption mutant has an impaired capacity for binding to Cascade. Based on this result, we concluded that the head domain of AcrIF24 is necessary for binding to Cascade. To confirm this conclusion, we purified a head domain deletion mutant (ΔHead) and analyzed the effect of the deletion on the interaction of AcrIF24 to Cascade. As expected, the ΔHead mutant could not bind to Cascade by failing to co-migrate with Cascade on SEC followed by SDS-PAGE (Figure 5E and F, and Supplementary Figure S5F). Based on these experiments, we confirmed that the head domain of AcrIF24 is necessary for binding to Cascade.

Next, we wondered which Cascade subunit(s) were critical for the interaction with AcrIF24. To answer this question, we purified each subunit of Cascade separately (Supplementary Figure S6) and performed SEC with a mixture of each subunit with AcrIF24 (Figure 5G–I); note that an interaction with Cas8f could not be tested due to insolubility. Although an apparent peak shift by forming a complex was not detected on the SEC profiles, AcrIF24 co-migrated with Cas7f1 but not with Cas5f and Cas6f on SDS-PAGE, indicating that AcrIF24 bound specifically to Cas7f1(Figure 5I). This result is in good agreement with recent a structural study published by Yang et al (35). The cryo-EM study of the complex between AcrIF24 and Cascade showed that AcrIF24 specifically binds to Cas7f1 when AcrIF24 inhibits the Cascade activity by direct binding.

### Dimerization and the AcrIF24 head are essential for inhibition of target DNA binding by I-F Cascade

**Figure 6.**
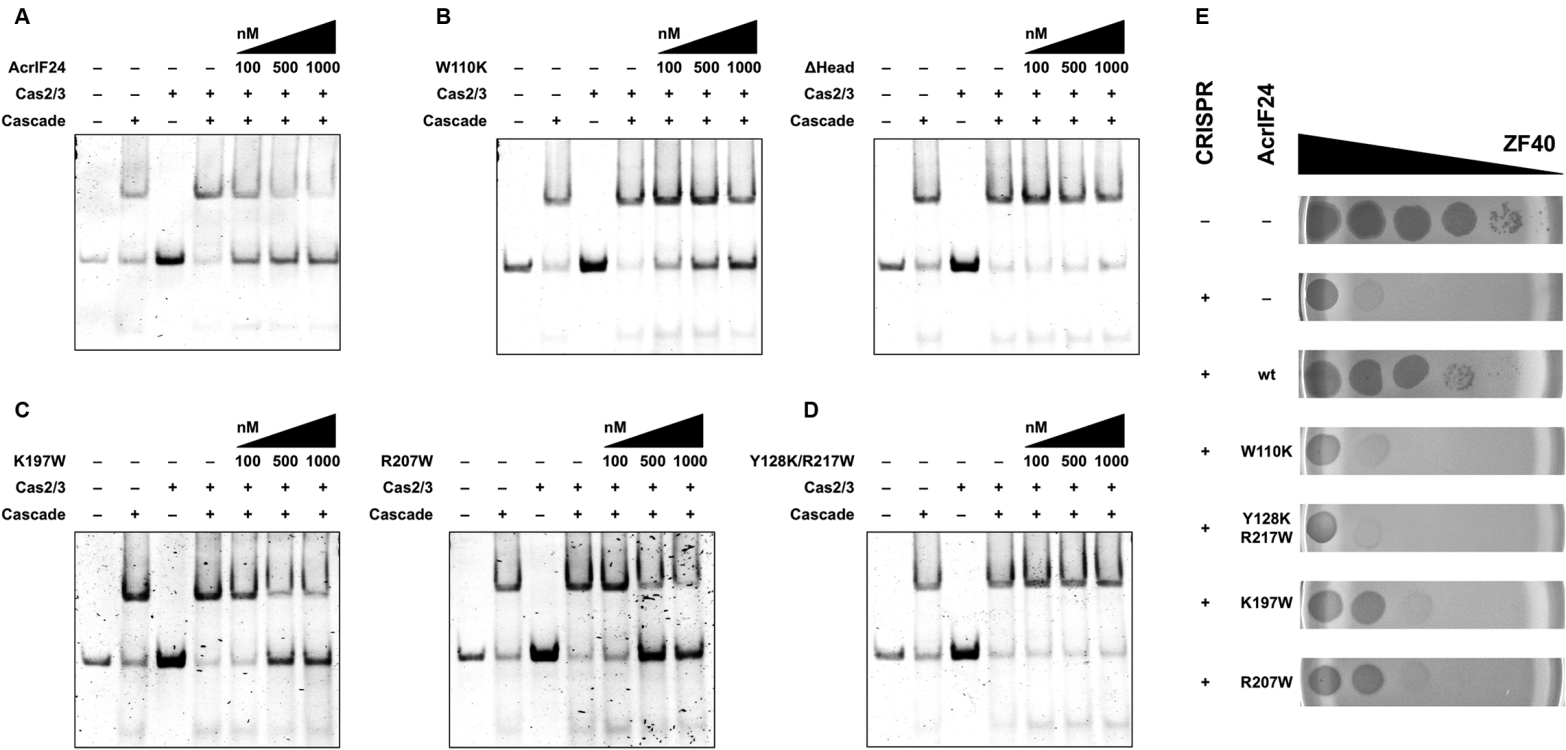
The head domain and dimerization of AcrIF24 are important for its anti-CRISPR activity. (**A**) EMSA performed with Cascade using target DNA as substrate. Purified wildtype AcrIF24 at the indicated concentrations was pre-mixed with Cascade and Cas2/3 before adding DNA substrate for analysing the anti-CRISPR function of AcrIF24. (**B**) EMSA for analysing the anti-CRISPR activity of two Cascade-binding disrupted mutants, W110K and ΔHead. (**C**) EMSA for analysing the anti-CRISPR activity of two promoter-binding disrupted mutants, K197Y and R207W. (**D**) EMSA for analysing the anti-CRISPR activity of dimer-disrupted mutant, Y128K/Y217W. Non-denaturing acrylamide gels stained with SYBR Gold are shown. Cas2/3 has been added in these assays but no particular role was detected. (**E**) Phage ZF40 infecting a sensitive (–CRISPR, PCF425) or immune (+CRISPR, PCF835) *P. carotovorum* host carrying either an empty vector (–AcrIF24, pBAD30) or a derived plasmid (pPF2964, pPF3482, pPF3483, pPF3484 or pPF3487) for production of the indicated AcrIF24 variants. ZF40 was added as spots from a 10-fold serial dilution, indicated by the back triangle.

We wanted to determine how AcrIF24 inhibits type I-F Cascade activity. Since many AcrIF proteins inhibit I-F Cascade binding to complementary dsDNA targets, we first tested whether this mechanism applies here. I-F Cascade was purified with a crRNA complementary to a target dsDNA. Addition of I-F Cascade to this probe led to binding, as observed by a shift on the EMSA (Figure 6A). The addition of Cas2/3 alone, or in combination with Cascade, had no effect on these reactions. Importantly, the addition of increasing concentrations of AcrIF24 reduced the quantity of bound dsDNA (Figure 6A). Therefore, AcrIF24 inhibits the ability of I-F Cascade to bind to its specific complementary invader targets. Cas2/3 was added in these assays but no particular role was detected in our assay.

Next, we asked which domains of AcrIF24 are important for its I-F Cascade inhibitory activity. We first tested the role of the head domain by examining the ability of the W110K and ΔHead AcrIF24 variants to inhibit Cascade. Although the W110K mutant still retained function, deletion of the entire head domain rendered AcrIF24 unable to inhibit Cascade function in dsDNA binding (Figure 6B). These findings are in agreement with our binding analysis of AcrIF24 with Cascade (Figure 5E and F), where ΔHead lost complete binding activity, while the W110K mutant still possessed some binding capability. Mutations that disrupted DNA binding and promoter repression by AcrIF24 (i.e. K197Y and R207W; Figure 3D and Figure 4) had no effect on inhibition of I-F Cascade (Figure 6C), suggesting that the DNA-binding (Aca) function of AcrIF24 may be independent from its Acr activity *in vitro*. Furthermore, AcrIF24 dimerization was required to inhibit I-F Cascade, since the Y128K/Y217W mutant failed to disrupt DNA binding by Cascade (Figure 6D).

Finally, we examined whether these *in vitro* results are also valid in an *in vivo* phage infection model. For this, we used *Pectobacterium carotovorum* RC5297, which has a type I-F CRISPR-Cas system, and an *acr*-less variant of phage ZF40. This phage efficiently infected *P. carotovorum* (-CRISPR), but infectivity was drastically reduced in the presence of a ZF40-targeting spacer in the host CRISPR-Cas system (+CRISPR, Figure 6E). However, even in the presence of a targeting spacer, overexpression of *acrIF24* from a plasmid allowed the phage to overcome CRISPR-Cas defense. In contrast, the W110K mutation completely abrogated the protective effect of AcrIF24 and therefore displayed a stronger phenotype *in vivo* than *in vitro*, highlighting the importance of this head domain residue for Acr activity (Figure 6E). The dimer-disruption mutations (Y128K/Y217W) also completely abrogated Acr activity, in agreement with our *in vitro* results, supporting that dimerization is important for inhibiting Cascade. Interestingly, the mutant proteins unable to repress the promoter (K197W and R207W) also slightly affected Acr function, albeit not as strongly as the W110K mutation in the head domain. This suggests that the Acr and Aca functions of AcrIF24 are not completely separable (Figure 3D). In summary, dimer formation and the head domain of AcrIF24 are critical for its Acr activity.

## DISCUSSION

The dual function of AcrIF24 as both an Acr and Aca was initially suggested by Pinilla-Redondo and colleagues (23). The gene encoding *P. aeruginosa* AcrIF24 was shown to confer anti-CRISPR activity and was in an operon lacking any *aca* gene. Part of the C-terminus of AcrIF24 was predicted as a HTH motif, which is a major DNA binding domain, suggesting that AcrIF24 contains Aca function in addition to anti-CRISPR activity (23). In this study, we solved the crystal structure of AcrIF24, which contains a novel domain composition reminiscent of a bird and consisting of head, body and wing domains. AcrIF24 is dimeric and binds a short inverted repeat sequence in the *acrIF23-acrIF24* operon promoter to repress *acr* expression. Through mutagenesis, we demonstrated that the body domain, which contains the HTH motif, was essential for DNA binding. We further show that AcrIF24 binds directly to the type I-F Cascade through interactions with the Cas7f1 subunit. Overall, our results uncover the structure and regulatory and anti-CRISPR activity of a dual function Acr-Aca protein.

AcrIF24 is a dimer through interactions between the head and body domains. Many DNA binding proteins with HTH motifs function as dimers. Indeed, dimerization of Aca proteins is critical for the recognition of inverted repeat (IR) DNA sequences in the promoter region of *acr* operons (52,55,58). In agreement, AcrIF24 bound to a short IR sequence in the promoter region of the *acr* operon and repressed transcription. The mechanism of action is likely due to blocking RNAP binding. While our manuscript was about to be submitted, another study on the structure of AcrIF24 was published (35). Interestingly, they were unable to solve the crystal structure of the complete protein, but of a deletion mutant with a linker instead of the head domain. This mutant dimerized still, despite lacking the head, even though the head appears to make important contributions to the protein-protein interface in our crystal structure. To understand operator recognition, both studies examined the roles of different amino acids in DNA binding. In terms of the body domain, we uncover roles of K197 and R207 for DNA binding, and Yang *et al.* show that R196 is also important (35). Because we show that an R16W mutation on the wing domain partially affected DNA binding, this domain might also contribute to promoter binding. The location of R16 at the bottom of AcrIF24 in line with the HTH motif supports the involvement of the wing in DNA recognition. Yang *et al.* show that their head domain deletion mutant still binds DNA, indicating this portion of AcrIF24 is not required for Aca activity. Interestingly, some AcrIF24 homologues are truncated, with the N-terminal wing domain missing, such as in *B. glumae* (Figure 3B). It is possible that AcrIF24 homologues evolved with or without the wing domain and that it provides an accessory DNA binding activity. Therefore, there are potentially two different classes of AcrIF24, a three domain version and a two domain version. The structure of AcrIF24 in complex with the IR DNA binding site will be critical for a detailed understanding of the exact DNA recognition strategy.

Diverse mechanisms of various anti-CRISPR proteins have been demonstrated (16,25,53,59). We showed that AcrIF24 directly binds to the type I-F Cascade complex with critical involvement of the head domain. Direct interactions of an Acr with Cascade can block recruitment of target DNA or the Cas3 nuclease and are the most common Acr strategies for inhibiting CRISPR-Cas systems (26,27). Among the twenty-four AcrIF family members identified, at least nine, including AcrIF1, AcrIF2, AcrIF4, AcrIF6, AcrIF7, AcrIF8, AcrIF9, AcrIF10 and AcrIF14, directly bind to Cascade for inhibition (26,27,60). Our results suggest that AcrIF24 also blocks the ability of I-F Cascade to bind target DNA to inhibit its CRISPR-Cas activity. This is in agreement with the recent cryoEM structure of dimeric AcrIF24 in complex with two Cascade complexes that was published while our manuscript was in preparation (35). Indeed, we showed that the mass of Cascade (∼400 kDa) was dramatically increased by complex formation with AcrIF24 (around 700–800 kDa). Furthermore, our *in vitro* and *in vivo* data show that dimerization is essential for AcrIF24 to inhibit Cascade. These results, together with the study by Yang *et al.* indicate that interaction of dimeric AcrIF24 with two Cascade complexes results in CRISPR-Cas inhibition.

Consistent with our study,*Yang et al*., showed that AcrIF24 binds to Cas7f1 subunits (Cas7.2f-Cas7.6f) in Cascade. Although most of AcrIF24 contributed to the Cas7 interaction, the major domain involved was the head. This is consistent with our mutagenesis studies showing reduced binding with a head mutant, and with our *in vivo* data showing complete loss of Acr activity in the W110K head-domain mutant. Furthermore, binding was completely abrogated when the head domain was deleted. Due to their ability to only solve the structure of the head via cryoEM in the AcrIF24-Cascade complex, the authors suggest that the head is disordered until interaction with Cascade. However, our intact, fully folded crystal structure of AcrIF24 indicates that AcrIF24 can be in ordered conformation without binding to Cascade. Indeed, superimposition of our AcrIF24 structure with AcrIF24 in complex with Cascade revealed identical structures except several loops on the head domain that are important for Cas7f recognition (Supplementary Figure S7A and B). This indicates that interaction with Cascade causes certain loops on, but not the entire, head domain to change conformation.

Given that Acrs are natural CRISPR-Cas inhibitors, their potential applications in bio-medical therapeutics, including antibacterial compounds, gene editing, and regulation of gene drives, have been suggested (2,16,61). Moreover, the engineering of Acr proteins for better usage has been intensively studied (62). In this context, the structural information of AcrIF24 and its dual function can contribute new information not only to the basic understanding of phage-host interactions and the field of CRISPR-Cas but also to potential bio-medical applications of Acrs.

## DATA AVAILABILITY

The coordinate and structure factor have been deposited into the Research Collaboratory for Structural Bioinformatics (RCSB) Protein Data Bank (PDB) under the PDB code of 7XI1.

## Supplementary Material

gkac880_Supplemental_File
