# Supplementary material for: Molecular basis of dual anti-CRISPR and auto-regulatory functions of AcrIF24
Source: Nucleic Acids Res. 2022 Oct 16;50(19):11344–58. doi: 10.1093/nar/gkac880 (PMC9638941; doi:10.1093/nar/gkac880)
Supplement: gkac880_Supplemental_File [file gkac880_supplemental_file.pdf]

Molecular basis of dual Anti-CRISPR and auto-regulatory functions of AcrIF24

Gi Eob Kim[1,2,†], So Yeon Lee[1,2,†], Nils Birkholz[3,4], Kotaro Kamata[3,4], Jae-Hee Jeong[5], Yeon-Gil Kim[5], Peter C. Fineran[3,4] and Hyun Ho Park[1,2,*]

[1]College of Pharmacy, Chung-Ang University, Seoul 06974, Republic of Korea

[2]Department of Global Innovative Drugs, Graduate School of Chung-Ang University, Seoul 06974, Republic of Korea

[3]Department of Microbiology and Immunology, University of Otago, PO Box 56, Dunedin 9054, New Zealand

[4]Bioprotection Aotearoa, University of Otago, PO Box 56, Dunedin 9054, New Zealand

[5]Pohang Accelerator Laboratory, Pohang University of Science and Technology, Pohang 790-784, Republic of Korea

[†] These authors contributed equally to this work.

*Correspondence to:
Hyun Ho Park; College of Pharmacy, Chung-Ang University, Seoul 06974, Republic of Korea; Tel: +82-2-820-5930; Fax: +82-2-820-3033; Email: xrayleox@cau.ac.kr

Supporting Information

A

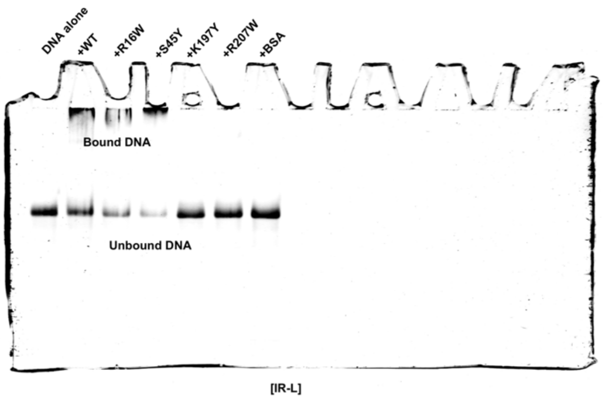

B

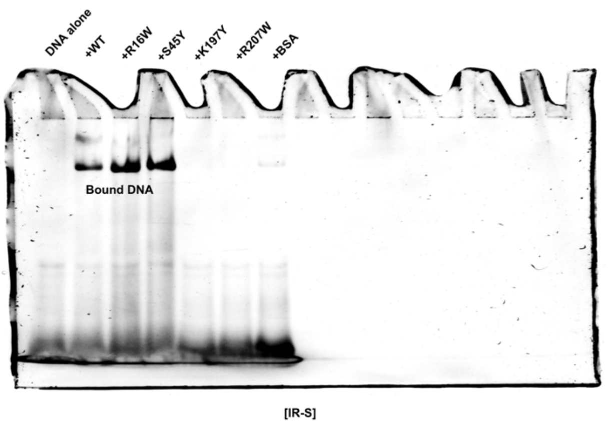

Supplementary Figure 1. Uncropped gels. Uncropped gel used for Fig. 4C upper panel (A) and lower panel (B).

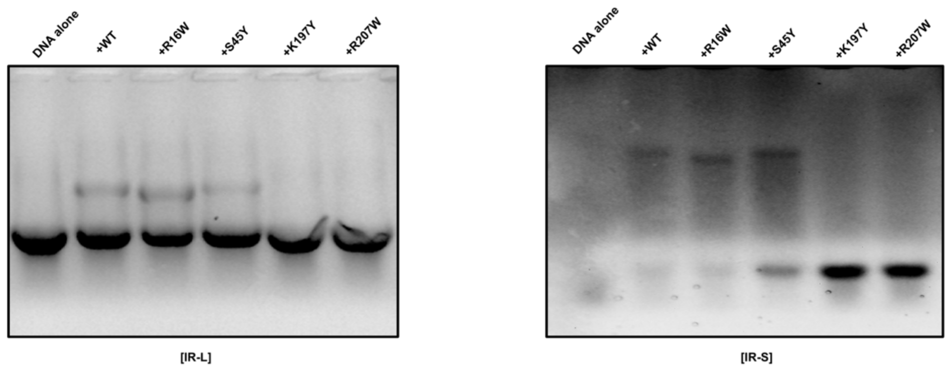

Supplementary Figure 2. Validation of the interaction of various AcrIF24 mutants with the promotor using EMSA on agarose gel.

A

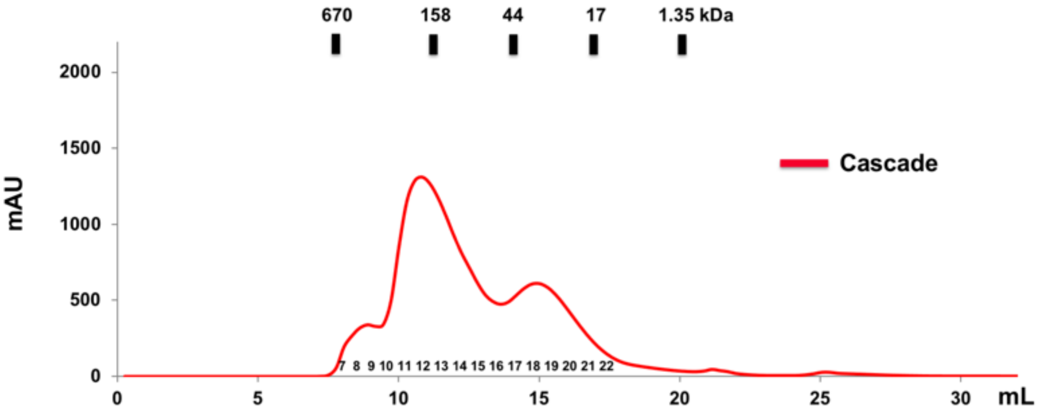

B

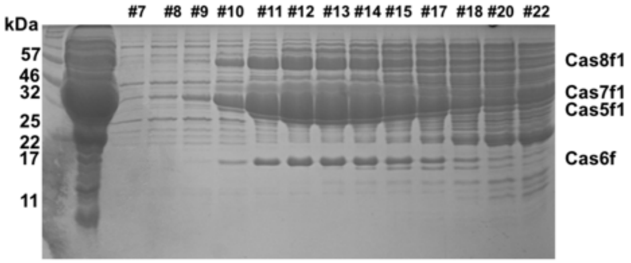

Supplementary Figure 3. Analysis of Cascade complex by SDS-PAGE following SEC. (A) SEC profile produced by a Cascade complex sample without AcrIF24. Fractions collected during SEC are numbered. (B) SDS-PAGE gel produced by loading the SEC fractions indicated above the gel.

A

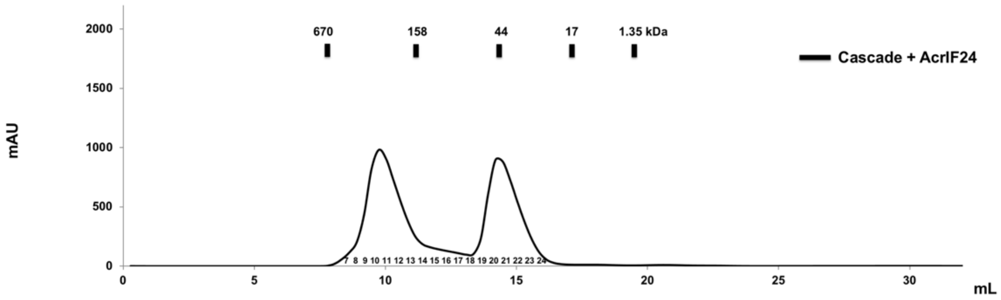

B

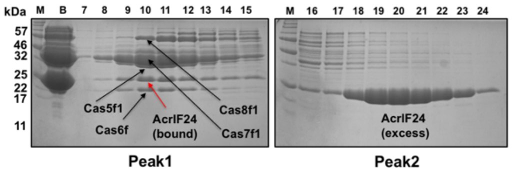

Supplementary Figure 4. Analysis of the mixture of Cascade complex and AcrIF24 on SEC followed by SDS-PAGE. (A) SEC profile produced by a Cascade complex sample with AcrIF24. Fractions collected during SEC are numbered. (B) SDS-PAGE gel produced by loading the SEC fractions indicated above the gel.

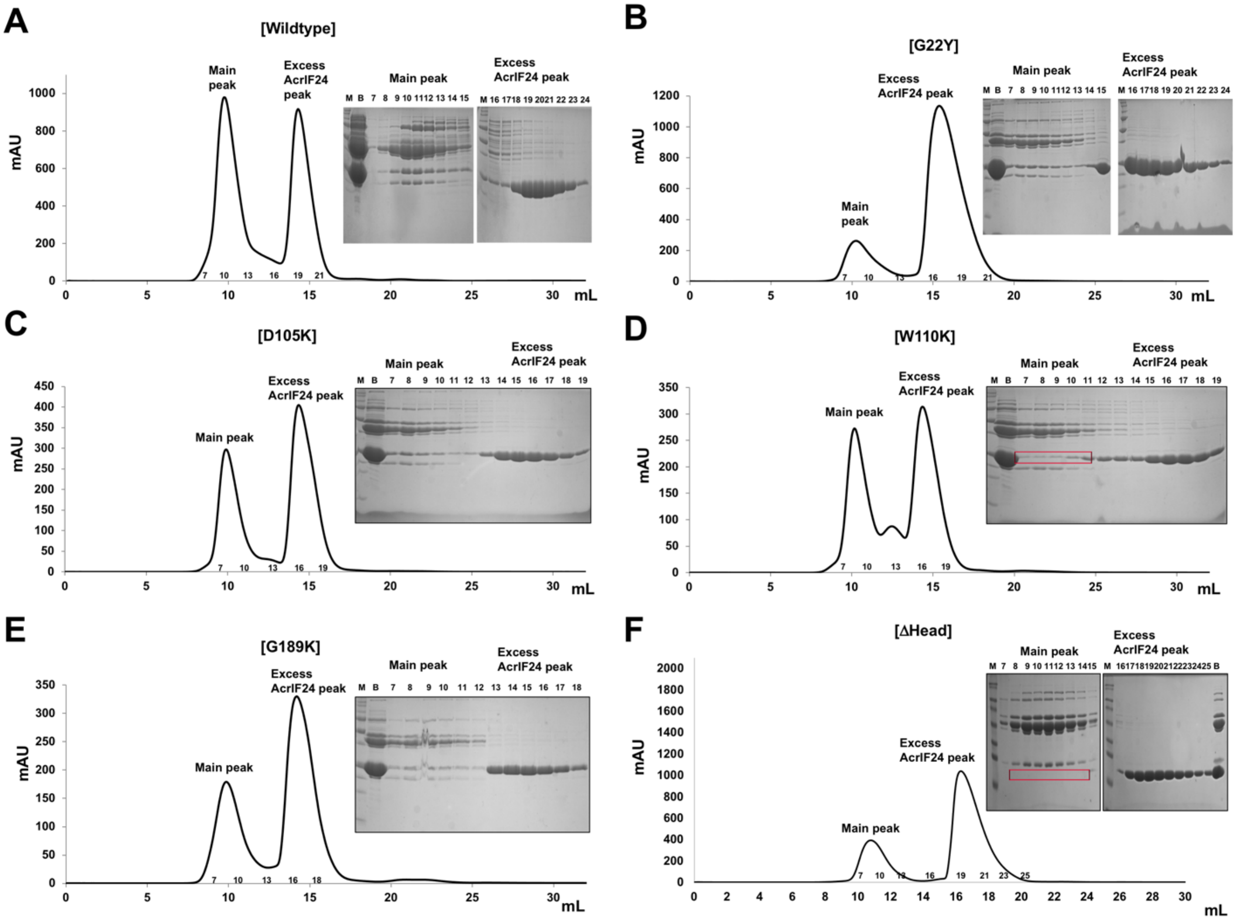

Supplementary Figure 5. Analysis of interaction between Cascade and various AcrIF24 mutants by SEC and SDS-PAGE. SEC and SDS-PAGE profiles of Cascade mixed with wild-type AcrIF24 (A) or various AcrIF24 mutants (B-F). SDS-PAGE gels with the main peak and excess AcrIF24 peak fractions are provided to the right of each SEC profile. In D and F, the reduced co-purification of AcrIF24 (W110K) and the absence of co-migration of AcrIF24 (ΔHead) with Cascade is indicated on the gel by a red box

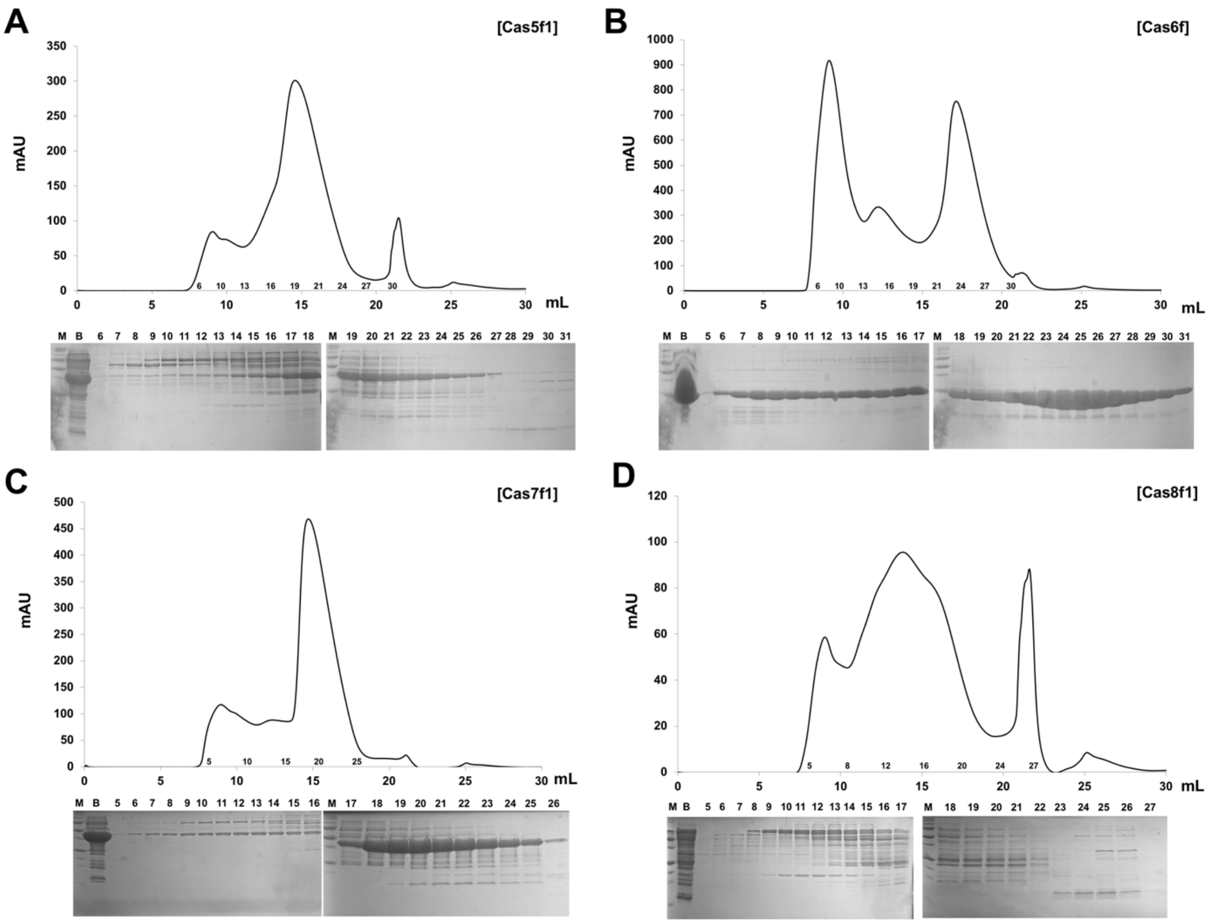

Supplementary Figure 6. Purification and analysis of Cascade components by SEC and SDS-PAGE. Protein samples for each Cascade component, Cas5f1 (A), Cas6f (B), Cas7f1 (C), and Cas8f1 (D) were loaded onto a SEC column, and peak and bands patterns were analyzed.

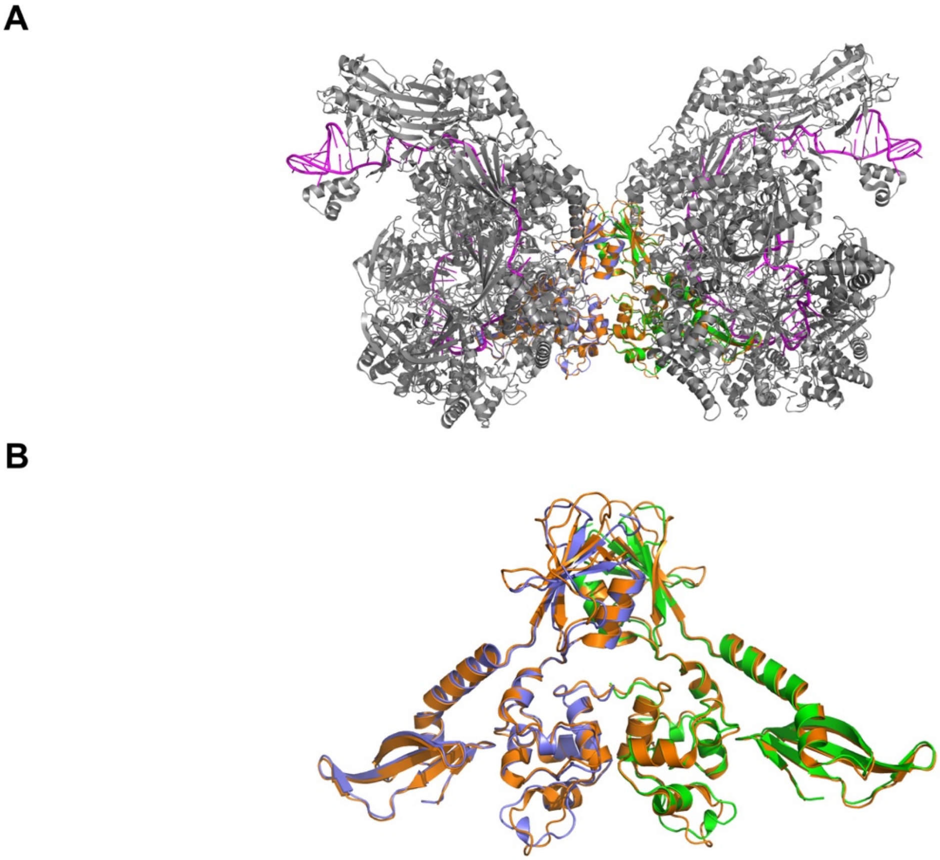

Supplementary Figure 7. Structural comparison of AcrIF24 alone with AcrIF24 in Cascade. (A) Superposition of the crystal structure of AcrIF24 solved in this study (monomers shown in green and blue; PDB: 7XI1) with the structure of AcrIF24 in complex with Cascade (PDB: 7ELM) solved by Yang et al. (2022). Gray and magenta color molecules indicate Cascade and crRNA, respectively. AcrIF24 in Cascade was shown in orange. (B) Magnified AcrIF24 structures used in panel A.

Supplementary Table1. Oligonucleotides used in this study.

Name	Sequence (5'→3')
AcrIF24-F	GGGCATATGATGAACGCGATTCATATTGG
AcrIF24-R	GGGCTCGAGGCTCGCGCGTTTCACGCCCA
Cas5-F	GGGCATATGATGAGCGTGACTGATCCCGA
Cas5-R	GGGCTCGAGTTATGCGATGGCGTGTTCGA
Cas6-F	GGGCATATGATGGACCACTACCTCGACAT
Cas6-R	GGGCTCGAGTCAGAACCAGGGAACGAAAC
Cas7-F	GGGCATATGATGAAATCTTCTCACCATCA
Cas7-R	GGGCTCGAGTTACTTCTCTTCGGCTTCAC
Cas8-F	GGGCATATGATGACCTCTCCCCTCCCAAC
Cas8-R	GGGCTCGAGTCAGTCACGCTCATCTTCGA
R16W(Mut)-sense	CATCACCCCGGCGGCGTGGGGCCTGCACTACGGTG
R16W(Mut)-antisense	CACCGTAGTGCAGGCCCCACGCCGCCGGGGTGATG
S45Y(Mut)- sense	CAAAACCCTGCCGGATTATTACACCTCTAGCGAA
S45Y(Mut)- antisense	TTCGCTAGAGGTGTAATAATCCGGCAGGGTTTTG
K197Y(Mut)- sense	GCGACCAACTTCCGTTATTACACCGCGGGCGAT
K197Y(Mut)- antisense	ATCGCCCGCGGTGTAATAACGGAAGTTGGTCGC
R207W(Mut)- sense	GTAAGCGCGGCGAACTGGCAGAAAATCTCTTTC
R207W(Mut)- antisense	GAAAGAGATTTTCTGCCAGTTCGCCGCGCTATC
IR-L-sense	CAGAATATCATTGCATAGCTCGATTCGAGCTAATATAGTAATACCAACAG CGCGGCACACCGCCAGCACTGAACCT
IR-L-antisense	AGGTTCAGTGCTGGCGGTGTGCCGCGCTGTTGGTATTACTATATTAGCT CGAATCGAGCTATGCAATGATATTCTG
IR-S-sense	TAGCTCGATTCGAGCTA
IR-S-antisense	TAGCTCGAATCGAGCTA
PF138	CACACTTTGCTATGCCATAG
PF139	GCTACTGCCGCCAGG
PF209	TCGTCTTCACCTCGAGAAATC
PF210	GTCATTACTGGATCTATCAACAGG
PF2910	TTTTGGTACCCGATGATCTGCCCGACATG
PF2911	TTTTGAGCTCCATGACGATGTATTGTTTAC
PF2914	CTGACCATCCGCATTTATCCGTCTCGCTTGCCCTGGA
PF2958	CTAGTCCAGGGCAAGCGAGACGGATAAATGCGGATGGTCAGCATG
PF2969	CGTTAGAGTGATCGGGCTAC
PF2970	CCTCAATCATTTGCTGATAATTGC
PF2971	CATATTCTCTTTAACAATCTGGTAGTTAGTG
PF2972	GTAATATCTACGCTGACGATGATG
PF5881	CACACTTTGCTATGCCATAGACTAGTCTGGTGGTCGAGTTTCACCGACC AGCCCTTGAATCGAATGACCAGCATGTCGCGCCCCTTGTAGGAAAACCC GCAGTGTACCGGCTCGACAGAATATCATTGCATAGCTCGATTCGAGCTA ATATAGTAATACCAACAGCGCGGCACACCGCCAGCACTGAACCTCCTCG GAGACGCAAAATGCATCCTGGCGGCAGTAGCTCGTCTTCACCTCGAGA AATCGAGCTCATGAACGCCATCCATATCGGGCCGTTCTCGATCACTCCG GCTGCCCGCGGACTGCATTACGGGGGCCTGCCGCATCACCAGTGGAC CCTATACTACGGGCCCCGGGAAATGGCGATAAAGACCCTACCGGACAG TTACACCTCGTCGGAGGTGAGGGACGAGTTCTCAGACATCATCGCCGA GTTCGTCATCGACGCCCGGCACCGATACGCGCCCGATGTTCTGGAACT GGTGAACTCGGATGGTGACGCAGTGCTCGCGCGAGTCGCCGTGAGCC GACTGCCAGAAGCGTTGTCCGGGTGCATACCAGACGATCGATTCCCATA CTGGCTCCTGACCGCCAGCCGCCCACGGCTCGGGCTCCCTGTCACCCT GAACGAGTACACCGCGCTCGCGGTCGAACTCAGCGCCCCTCCACTTGC

	ATGGATCACAGGGCTCCTCCCTGGCGAGGTACTGACACATGACGCCGA<br>GGAGTGGCGACCGCCGACCAGTTGGGAGCTACGCCACGTTGTCGGCG<br>AGGGGTCGTTTACTGGCGTAAGCGGCGCCGCTGCGGCCGCTCTGCTC<br>GGAATGTCCGCAACGAATTTCCGAAAGTACACAGCCGGGGACTCTGCC<br>GCGAATCGCCAGAAAATCAGTTTCGCAGCCTGGCACTACCTACTCGACC<br>GGCTCGGCGTGAAGCGGGCGAGCTGAGCATGCCCTGTTGATAGATCCA<br>GTAATGAC
PF6867	TCCCATACAAGCTCCTGACCGCCAGCCG
PF6868	GGAGCTTGTATGGGAATCGATCGTCTGGTATGCACC
PF6869	AATTTCCGATACTACACAGCCGGGGACTCTG
PF6870	GTAGTATCGGAAATTCGTTGCGGACATTCCGAG
PF6871	GCGAATTGGCAGAAAATCAGTTTCGCAGCCTGG
PF6872	TTTCTGCCAATTCGCGGCAGAGTCCCCGG
PF6976	TCGTCTTCACCTCGAGAAATCGAGCTCATGAACGCCATCCATATCGGGC<br>CGTTCTCGATCACTCCGGCTGCCCGCGGACTGCATTACGGGGGCCTGC<br>CGCATCACCAGTGGACCCTATACTACGGGCCCCGGGAAATGGCGATAA<br>AGACCCTACCGGACAGTTACACCTCGTCGGAGGTGAGGGACGAGTTCT<br>CAGACATCATCGCCGAGTTCGTCATCGACGCCCGGCACCGATACGCGC<br>CCGATGTTCTGGAACTGGTGAACTCGGATGGTGACGCAGTGCTCGCGC<br>GAGTCGCCGTGAGCCGACTGCCAGAAGCGTTGTCCGGGTGCATACCAG<br>ACGATCGATTCCCATACTGGCTCCTGACCGCCAGCCGCCCACGGCTCG<br>GGCTCCCTGTCACCCTGAACGAGAAGACCGCGCTCGCGGTCGAACTCA<br>GCGCCCCTCCACTTGCATGGATCACAGGGCTCCTCCCTGGCGAGGTAC<br>TGACACATGACGCCGAGGAGTGGCGACCGCCGACCAGTTGGGAGCTAC<br>GCCACGTTGTCGGCGAGGGGTCGTTTACTGGCGTAAGCGGCGCCGCT<br>GCGGCCGCTCTGCTCGGAATGTCCGCAACGAATTTCCGAAAGTACACA<br>GCCGGGGACTCTGCCGCGAATCGCCAGAAAATCAGTTTCGCAGCCTGG<br>CACTGGCTACTCGACCGGCTCGGCGTGAAGCGGGCGAGCTGAGCATG<br>CCCTGTTGATAGATCCAGTAATGAC
Y128K(Mut)-sense	GTT ACC CTG AAC GAA AAA ACC GCG CTG GCT GTT
Y128K(Mut)-antisense	AAC AGC CAG CGC GGT TTT TTC GTT CAG GGT AAC
Y217W(Mut)-sense	CTT TCG CGG CGT GGC ACT GGC TGC TGG ATC GTC TGG GC
Y217W(Mut)-antisense	GCC CAG ACG ATC CAG CAG CCA GTG CCA CGC CGC GAA AG
T129W(Mut)-sense	CGG TTA CCC TGA ACG AAT ACT GGG CGC TGG CTG TTG AAC TGT C
T129W(Mut)-antisense	GAC AGT TCA ACA GCC AGC GCC CAG TAT TCG TTC AGG GTA ACC G
R221W(Mut)-sense	CAC TAC CTG CTG GAT TGG CTG GGC GTT AAA CGT
R221W(Mut)-antisense	ACG TTT AAC GCC CAG CCA ATC CAG CAG GTA GTG
ΔHead	AACGCGATCCACATCGGCCCGTTCAGCATCACCCCGGCGGCGC<br>GTGGCCTGCACTACGGTGGCCTGCCGCACCACCAGTGGACCCT<br>GTACTACGGCCCGCGTGAAATGGCGATCAAAACCCTGCCGGATT<br>CTTACACCTCTAGCGAAGTGCGTGATGAATTCAGCGACATCATCG<br>CGGAATTCGTGATCGATGCGCGTCACCGTTACGCGGGCGGCGG<br>CTCTGCGCCGCCGCTGGCGTGGATCACCGGTCTGCTGCCGGGT<br>GAAGTTCTGACCCACGATGCGGAAGAATGGCGTCCGCCGACCTC<br>TTGGGAACTGCGTCACGTTGTTGGTGAAGGCTCTTTCACCGGCG<br>TTAGCGGCGCGGCGGCGGCGGCGCTGCTGGGCATGAGCGCGA<br>CCAACTTCCGTAAATACACCGCGGGCGATAGCGCGGCGAACCGT<br>CAGAAAATCTCTTTCGCGGCGTGGCACTACCTGCTGGATCGTCT<br>GGGCGTTAAACGTGCGAGC
